# Complex Behavior of ALDH1A1 and IGFBP1 in Liver Metastasis from a Colorectal Cancer

**DOI:** 10.1371/journal.pone.0155160

**Published:** 2016-05-06

**Authors:** Jin Cheon Kim, Ye Jin Ha, Ka Hee Tak, Seon Ae Roh, Chan Wook Kim, Tae Won Kim, Seon-Kyu Kim, Seon-Young Kim, Dong-Hyung Cho, Yong Sung Kim

**Affiliations:** 1 Department of Surgery, University of Ulsan College of Medicine, Seoul, Korea; 2 Department of Medicine, University of Ulsan College of Medicine, Seoul, Korea; 3 Institute of Innovative Cancer Research, Asan Medical Center, Seoul, Korea; 4 Medical Genomics Research Center, Korea Research Institute of Bioscience & Biotechnology, Daejeon, Korea; 5 Graduate School of East-West Medical Science, Kyung Hee University, Gyeonggi-do, Korea; University of Alabama at Birmingham, UNITED STATES

## Abstract

Using our data set (GSE50760) previously established by RNA sequencing, the present study aimed to identify upregulated genes associated with colorectal cancer (CRC) liver metastasis (CLM) and verify their biological behavior. The potential roles of candidate genes in tumors were assessed using cell proliferation and invasion assays. Tissue samples were collected from 18 CRC patients with synchronous CLM and two CRC cell lines (SW480 and SW620) were used for transfection and cloning. The roles of the genes identified in CLM were verified using immunohistochemistry in 48 nude mice after intrasplenic transplantation of CRC cells. mRNA and protein expression was determined by quantitative real-time reverse transcription polymerase chain reaction and western blot, respectively. Nine genes were initially selected according to the relevance of their molecular function and biological process and, finally, *ALDH1A1* and *IGFBP1* were chosen based on differential mRNA expression and a positive correlation with protein expression. The overexpression of ALDH1A1 and IGFBP1 significantly and time-dependently decreased cell proliferation (*p* ≤ 0.001–0.003) and suppressed invasiveness by ≥3-fold over control cells (*p* < 0.001) in the SW480 cell line, whereas they had a slight effect on reducing SW620 cell proliferation. The protein expression levels of E-cadherin, N-cadherin, claudin-1, and vimentin were significantly higher in CLM than in primary tumor tissues (*p* < 0.05). However, the cadherin switch, namely, N-cadherin overexpression with reduced E-cadherin expression, was not observed in CLM tissues and transfected CRC cells. Irrespective of reduced proliferation and invasion found on *in vitro* cell assays, persistent overexpression of β-catenin, vimentin, and ZO-1 in IGFBP1-overexpressing SW480 cells possibly contributed to CLM development in mice implanted with IGFBP1-overexpressing SW480 cells (CLM occurrences: SW480/*IGFBP1*-transfected mice *vs*. SW480/vector- and SW480/*ALDH1A1*-transfected mice, 4/8 *vs*. 0/10, *p* = 0.023). In conclusion, ALDH1A1 and IGFBP1 are differentially overexpressed in CLM and may play a dual role, functioning as both tumor suppressors and metastasis promoters in CRC.

## Introduction

Liver metastasis frequently occurs in colorectal cancer (CRC), resulting in the survival of disseminated tumor cells in the liver. Tumor cells that escape from the primary tumor and reach a metastatic site interact with the microenvironment [1]. In liver metastasis of CRC (CLM), the fate of tumor cells is primarily determined by their interactions with hepatic sinusoidal/extra-sinusoidal cells [2]. Hepatic stellate cells play a major role in CLM by releasing various factors that promote CLM, including growth factors [transforming growth factor-β (TGF-β), epidermal growth factor, vascular endothelial growth factor, and insulin-like growth factor (IGF)-I] and metalloproteinases [3]. The six members of the IGF-binding protein (IGFBP) family were initially characterized as passive reservoirs of circulating IGFs but were later shown to play diverse roles in intracellular and pericellular compartments in the regulation of cell growth and survival [4]. However, previous studies that investigated the relationships between altered serum IGFBP levels and the presence or risk of various cancers had inconclusive and contradictory results [4,5].

On the other hand, aldehyde dehydrogenase 1A1 (ALDH1A1), one of 19 ALDH isoforms, affects the ALDH activity of cancer stem cells (CSCs). ALDH1A1 levels appear to be positively correlated with the prognosis of various cancers, although a combined assessment may better improve their prognostic potential [6]. Concurrently, because ALDH1A1 plays a particular role in detoxifying cyclophosphamide class chemotherapeutic agents, ALDH1A1 suppression possibly sensitizes colon CSCs to these regimens.

RNA-Seq technology provides abundant qualitative transcriptome information. However, valuable data sets need to be maximally used to extract candidate molecules according to specific biological endpoints by using adequately stratified computational and experimental tools. Because mRNA and protein expression data are complementary, concurrent measurement of both provides a better understanding of the biology of complex systems [7]. Meanwhile, biological replicates are essential in RNA-Seq experiments to draw generalized conclusions regarding the differences between two or more groups [8].

Because some genes have dual functions, such as both oncogenic and tumor-suppressive, it needs to be biologically verified whether candidate molecules associated with CLM promote or inhibit tumor progression. For example, the protective nature of autophagy has a dual effect on cancer, acting as a tumor suppressor in the early stages of tumorigenesis but supporting cancer progression in established tumors [9]. Similarly, the oncoprotein c-Myc can concurrently induce tumors with high frequency and massive programmed cell death in most transgenic mouse models [10]. TGF-β signaling is another example of a molecule with a dual effect, acting as both tumor suppressor and promoter [11]. In mouse models, several solid cancers, including CRC, revealed a biphasic function for TGF-β, whereby it inhibits the initial stage of tumorigenesis but subsequently boosts malignant progression and metastasis.

The primary aim of our present study was to use RNA sequencing to select significantly upregulated genes associated with CLM. We verified their biological behavior and determined whether the selected genes were implicated in CLM using matched tissue samples (normal colonic epithelium, primary tumor, and liver metastasis) from the same subject and an *in vivo* animal model.

## Materials and Methods

### Initial screening of CLM-related genes from the data set

**Fig 1:**
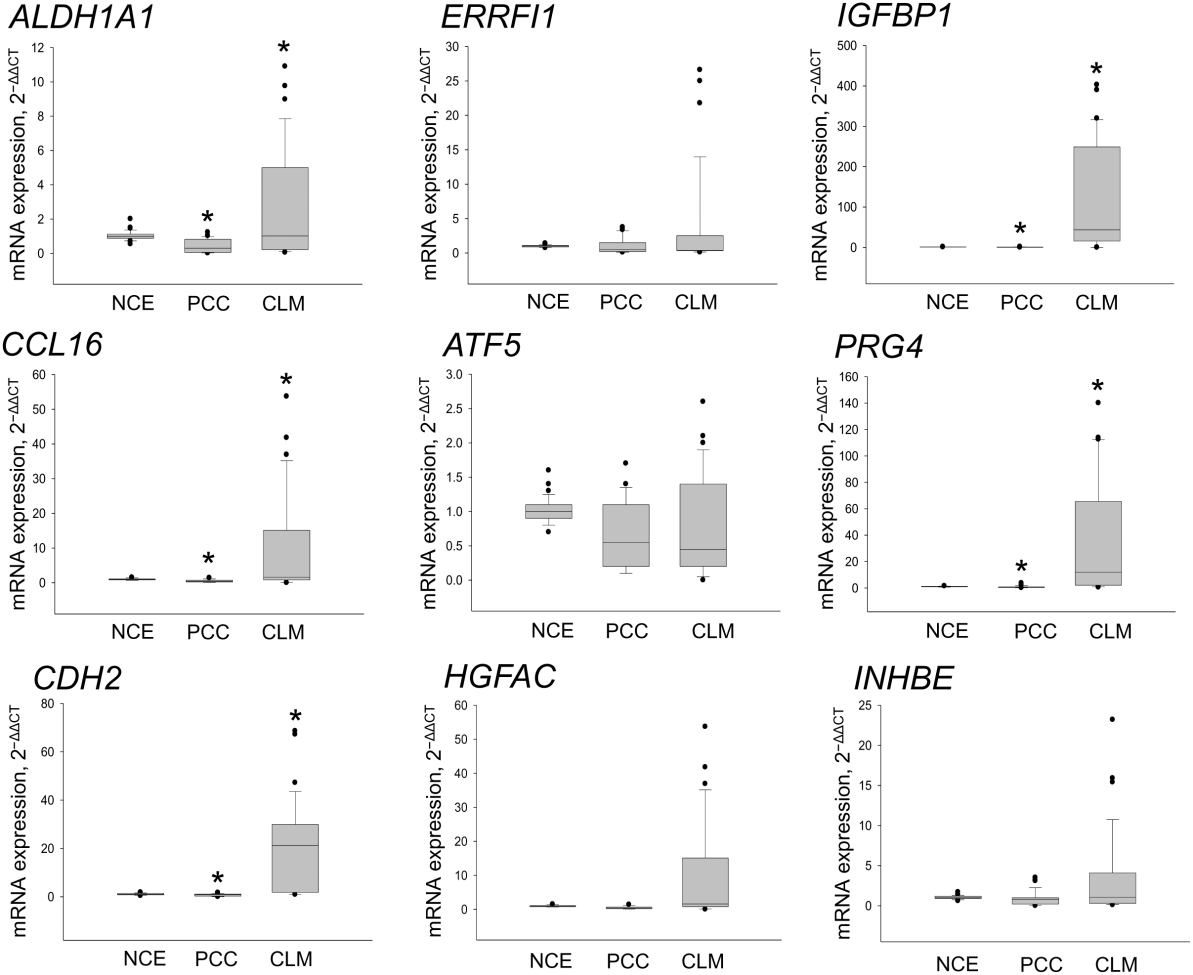
The mRNA expressions of the nine genes in ten CLM patients. Six genes showed differences in expression level of more than 1-fold in PCC (2^ΔCtPCC-ΔCtNCE^) compared with CLM (2^ΔCtCLM-ΔCtNCE^) (indicating upregulation of relevant genes). *p ≤ 0.001 between PCC and CLM. CLM, colorectal cancer liver metastasis; PCC, primary colorectal cancer.

The study protocol was approved by the Institutional Review Board for Human Genetic and Genomic Research of the Asan Medical Center, Seoul, Korea (registration no. 2014–0150). All participants provided their written informed consent. This study was also reviewed and approved by the Institutional Animal Care and Use Committee of the Asan Institute for Life Sciences, Seoul, Korea (registration no. 2014-03-055). The committee abides by the institute of Laboratory Animal Resources (ILAR) guide. A data set previously generated by RNA-Seq and available in the NCBI Gene Expression Omnibus public database under the accession number GSE50760 was used [12]. Tissue samples were collected from 18 CRC patients with synchronous CLM during the RNA-Seq assay and from an additional 10 patients for the current study (S1 Table). All patients had CRC with synchronous liver metastasis. Patients were excluded if they had a previous history of any cancer, concurrent cancer, hereditary CRC, or inflammatory bowel disease. Individual tissue samples consisted of matched normal colonic epithelium (NCE; >5 cm from the tumor border), primary CRC (PCC), and liver metastases (CLM) histologically identified as adenocarcinoma. The mRNA expression was compared between PCC and CLM by RNA-Seq to select 998 genes with ≥2-fold changes in expression based on a GLM likelihood ratio test (*p* < 0.001) (S2 Table) [13]. After the exclusion of 309 liver-specific genes based on the TiGER database [14], 689 genes were further filtered out to select 97 genes that were consistently upregulated in >50% of the CRC patients examined (S3 Table). These genes were finally narrowed down to nine genes according to the relevance of their molecular function and biological process [15], namely, cell growth and proliferation, extracellular matrix, epithelial-mesenchymal transition (EMT), and/or cancer stem cell, angiogenesis, chemotaxis, and apoptosis, as determined by the Gene Ontology Consortium (http://geneontology.org): *IGFBP1*, hepatocyte growth factor activator (*HGFAC*), chemokine ligand 16 (*CCL16*), inhibin beta E (*INHBE*), activating transcription factor 5 (*ATF5*), proteoglycan 4 (*PRG4*), cadherin 2 type 1 (*CDH2*), *ALDH1A1*, and ERBB receptor feedback inhibitor 1 (*ERRFI1*) (Fig 1).

### RNA isolation and real time reverse transcription-PCR

Total RNA was extracted from patient samples and cell lines using TRIzol^®^ Reagent (Invitrogen, Carlsbad, CA) according to the manufacturer’s instructions. The cDNA was synthesized from total RNA by amplification using random primers and SuperScript II RT (Invitrogen). Primers for target genes are listed in S4 Table. Glyceraldehyde 3-phosphate dehydrogenase (*GAPDH*) was used as an internal control. Quantitative real-time reverse transcription polymerase chain reaction (RT-PCR) was performed on a LightCycler 96 using the SYBR Green I Master Mix (Roche, Mannheim, Germany). The cycling reaction was started with pre-incubation at 95°C for 10 min, followed by 45 cycles of amplification (95°C for 10 sec, the Tm for 10 sec, and 72°C for 10 sec). The melting procedure included three conditions (95°C, 65°C, and 97°C for 10 sec), and cooling was finally performed at 37°C for 30 sec. The relative level of gene expression was determined using the –ΔΔCt method [16]. The Ct value was defined as the threshold PCR cycle when the amplified product was first detected.

### Colorectal cell lines, transfection, and cloning

The 10 CRC cell lines (DLD-1, HCT116, HCT15, HT29, LoVo, LS174T, RKO, SW480, SW620, and WIDR), two normal colonic cell lines (CCD-18Co and CCD841), and 3T3 fibroblasts were purchased from the American Type Tissue Culture Collection (Manassas, VA) and cultured in RPMI-1640 supplemented with 10% (v/v) fetal bovine serum and 1% (w/v) penicillin and streptomycin following the provider’s recommendations. The relative mRNA expressions of *ALDH1A1* and *IGFBP1* were negligible compared with *GAPDH* expressions in SW480 cells (cloned from primary CRC) and SW620 cells (cloned from the metastatic lymph nodes of the same subject) (S1A Fig). *ALDH1A1* and *IGFBP1* cDNAs (Origene, Rockville, MD) were amplified by PCR and subcloned into DDK-tagged pCMV6-Entry for stable transfection (Origene). Transient transfection was performed into SW480 and SW620 CRC cells using Lipofectamine 2000 (Invitrogen). Control cell lines were established by empty vector transfection. ALDH1A1- or IGFBP1-expressing cells were generated by G418 selection for 10 days, selecting at least two clones for each cell line. Stable expressions of ALDH1A1 and IGFBP1 were confirmed by western blot analysis, as previously described [17].

### Western blotting and immunohistochemistry (IHC)

Proteins were extracted from cultured cells using cell lysis buffer (Cell Signaling, Beverly, MA, USA). Equal amounts of proteins were separated by SDS-PAGE and transferred to PVDF membranes (Millipore, Billerica, MA, USA), which were blocked in 5% skim milk in TBST. Membranes were then incubated with primary antibody and HRP-conjugated secondary antibodies [anti-ALDH1A1, anti-phospho-FAK (Tyr397), anti-FAK, anti-survivin, anti-β-catenin, anti-Myc antibodies, and EMT antibody sampler kit (Cell Signaling); anti-IGFBP1, anti-IGFBP1, anti-CD44, and anti-CD166 antibodies (Abcam, Cambridge, UK), anti-CD133 antibody (MyBioSource, San Diego, California, USA)]. The specific complexes were detected using a SuperSignal West Pico kit (Thermo Scientific, Rockford, IL, USA). Data were quantified and analyzed using a GS-800 calibrated densitometer (Bio-Rad, Hercules, CA, USA). Relative band intensity values were calculated by normalizing the experimental absolute intensity to that of the corresponding β-actin band as a loading control. Additionally, five CLM samples in paraffin blocks were randomly selected from the 18 patients of the initial RNA-Seq to examine the degree of contamination of normal liver tissues by IHC using Heppar-1 and CK-7 (DAKO, Carpinteria, CA, USA) monoclonal antibodies to hepatocytes and bile duct cells, respectively, as previously described [17] (S2 Fig).

### Cell proliferation and cell cycle distribution assays

Control and treated SW480 and SW620 CRC cells were seeded onto 96-well plates. The proliferation rate was measured daily for 5 days using a cell proliferation assay kit (CCK-8: Dojindo, Kumamoto, Japan) on a microtiter plate reader adjusted to measure absorbance at 450 nm (Tecan, Melbourne, Australia). For flow cytometry assays, 5 × 10^5^ cells were suspended in ice-cold phosphate-buffered saline (PBS), fixed with 70% ethanol for 1 h, and incubated with propidium iodide solution (50 μg/mL) (Sigma, St Louis, MO, USA) for 30 min. After washing with PBS, 10,000 fluorescent cells for each sample were analysed by the FACSCalibur system (Becton Dickinson, Heidelberg, Germany) using flow cytometric system software (Becton Dickinson).

### Invasion and gelatin zymography assays

Control and treated SW480 and SW620 CRC cells (2 × 10^5^ cells each) were seeded onto the upper chamber of 24-well culture plates for matrigel invasion assays (BD Biocoat^™^: BD Biosciences, San Jose, CA, USA) according to the manufacturer’s guidelines. The 3T3-fibroblast-conditioned medium was placed in the lower chamber as a chemoattractant. After incubation at 37°C for 24 h, cells on the upper surface of the filter were completely wiped out and filters were stained with 0.2% crystal violet for 10 min. Cells attached to three different fields were counted under a light microscope (×100), and all assays were performed in triplicate. Matrix metalloprotease (MMP)-2 and MMP-9 activities in the culture media were examined by gelatin zymography. Aliquots of 10× concentrated conditioned media were mixed with sample buffer and electrophoresed on a 10% sodium dodecyl sulfate-polyacrylamide gel with 0.1% gelatin (Invitrogen) incorporated as a substrate for gelatinolytic proteases under non-reducing conditions at 125 V for 2 h. Gels were incubated at 37°C for 16 h in a fresh developing buffer and stained with 0.5% Coomassie brilliant blue R-250 (Bio-Rad). Bands on the gels were quantified using a densitometer.

### *In vivo* transplantation, growth, and metastasis of CRC cells

We used eight mice (6-week-old BALB/c-SLc-nu: Japan SLC, Shizuoka, Japan) for each of the six groups with different CRC cell transplantation, *i*.*e*., SW480/vector, SW480/ALDH1A1, SW480/IGFBP1, SW620/vector, SW620/ALDH1A1, and SW620/IGFBP1. A total of 5 × 10^6^ CRC cells were transplanted into the spleen, and mice were bred for 12 weeks, after which liver metastasis was identified by PET-MRI imaging using a sequential animal imaging system (NanoScan PET/MRI: Mediso, Budapest, Hungary). Animals were sacrificed to examine transplanted and metastatic tumors, concurrently measured using digital calipers (Mitutoyo, Kanagawa, Japan). All samples were histologically identified by hematoxylin and eosin (H & E) staining.

### Statistical Analysis

Demographic and biological features between the two groups and CLM occurrences among the *in-vivo* transplantation groups were appropriately compared using Fisher’s exact test or unpaired Student’s *t*-test, as appropriate. Pearson’s correlation test was used to assess a relationship between mRNA and protein expressions. Differential expressions of mRNA and cellular activity assays between the two groups were compared by Mann-Whitney’s *u*-test. Statistical significance was assigned when the *p*-values were <0.05. All calculations were performed using SPSS software (ver.21, SPSS Inc., Chicago, IL, USA).

## Results

### Candidate genes possibly associated with CLM

**Fig 2:**
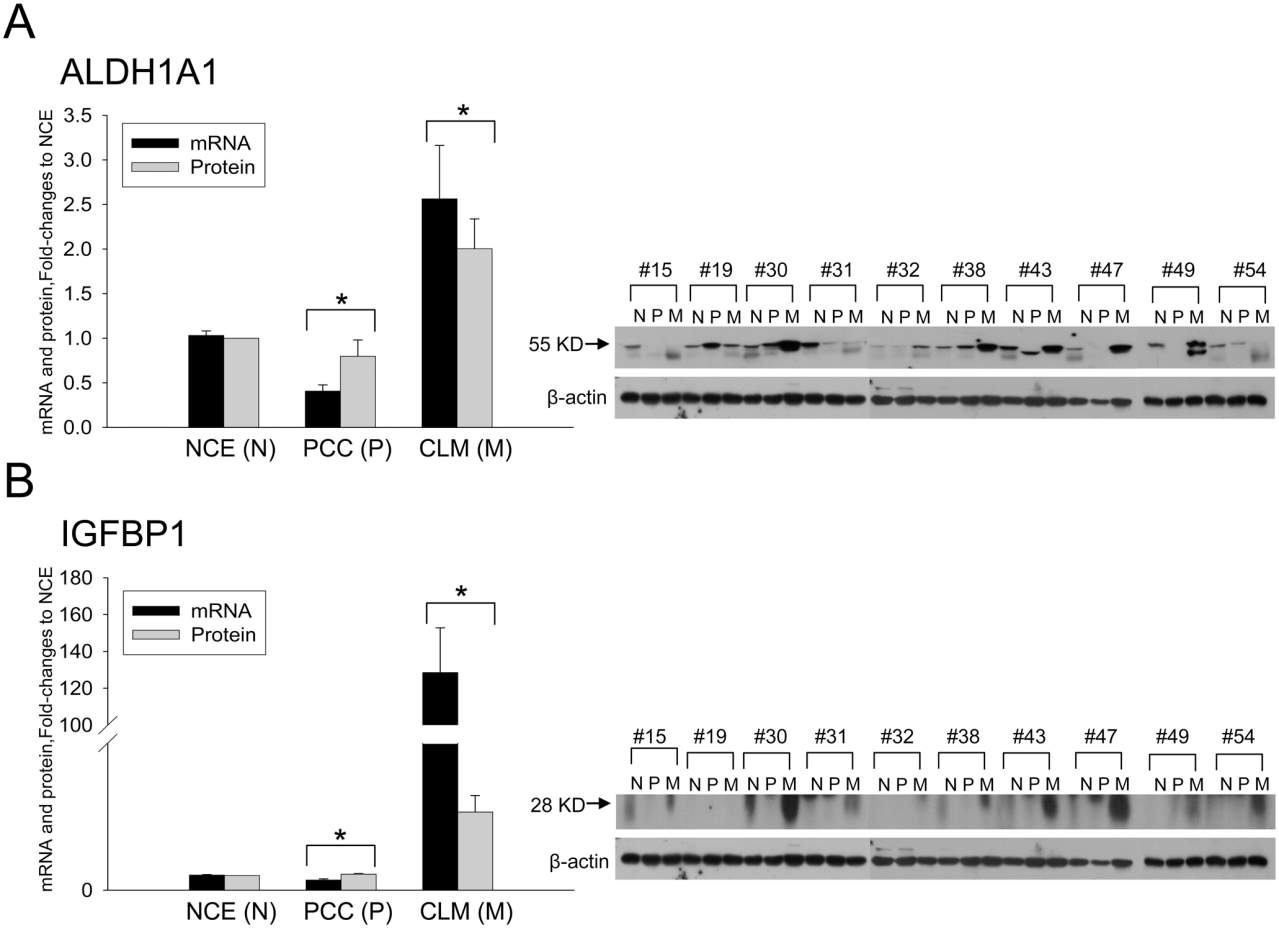
The mRNA and protein expressions of ALDH1A1and IGFBP1 in ten CLM patients. The expression levels of *ALDH1A1* (A) and *IGFBP1* (B) were higher in CLM than in PCC tissues (specifically in 4 patients: #30, 38, 43, 47). *p < 0.001–0.05 between PCC and CLM. NCE (N), normal colic epithelium; PCC (P), primary colorectal cancer; CLM (M), colorectal cancer liver metastasis.

The relative mRNA expression of the nine candidate genes was assessed in the 10 CRC patients with synchronous CLM (Fig 1). The relative mRNA expression was significantly higher in CLM (2^ΔCtCLM-ΔCtNCE^) than in PCC (2^ΔCtPCC-ΔCtNCE^) samples, and the expression of six genes differed by >1-fold (indicating upregulation of relevant genes) (*p* ≤ 0.001–0.003). Among these six genes, the mRNA expression levels of *ALDH1A1* and *IGFBP1* were closely correlated with their protein expression values in PCC and CLM tissues in the respective patient (*r* = 0.496 and *p* < 0.001, respectively) and concurrently showed higher expression in CLM than PCC tissues (*p* < 0.001–0.05) (Fig 2). Thus, ALDH1A1 and IGFBP1 were selected for further biological assays. Our CLM samples showed >97–99% tumor cell homogeneity on IHC, and the mean fold increases in ALDH1A1 and IGFBP1 after normalization to β-actin expression were 6.2 and 3.1, respectively, relative to the adjacent normal liver tissues (S2 Fig).

### Overexpression of ALDH1A1 and IGFBP1 inhibits CRC cell proliferation

**Fig 3:**
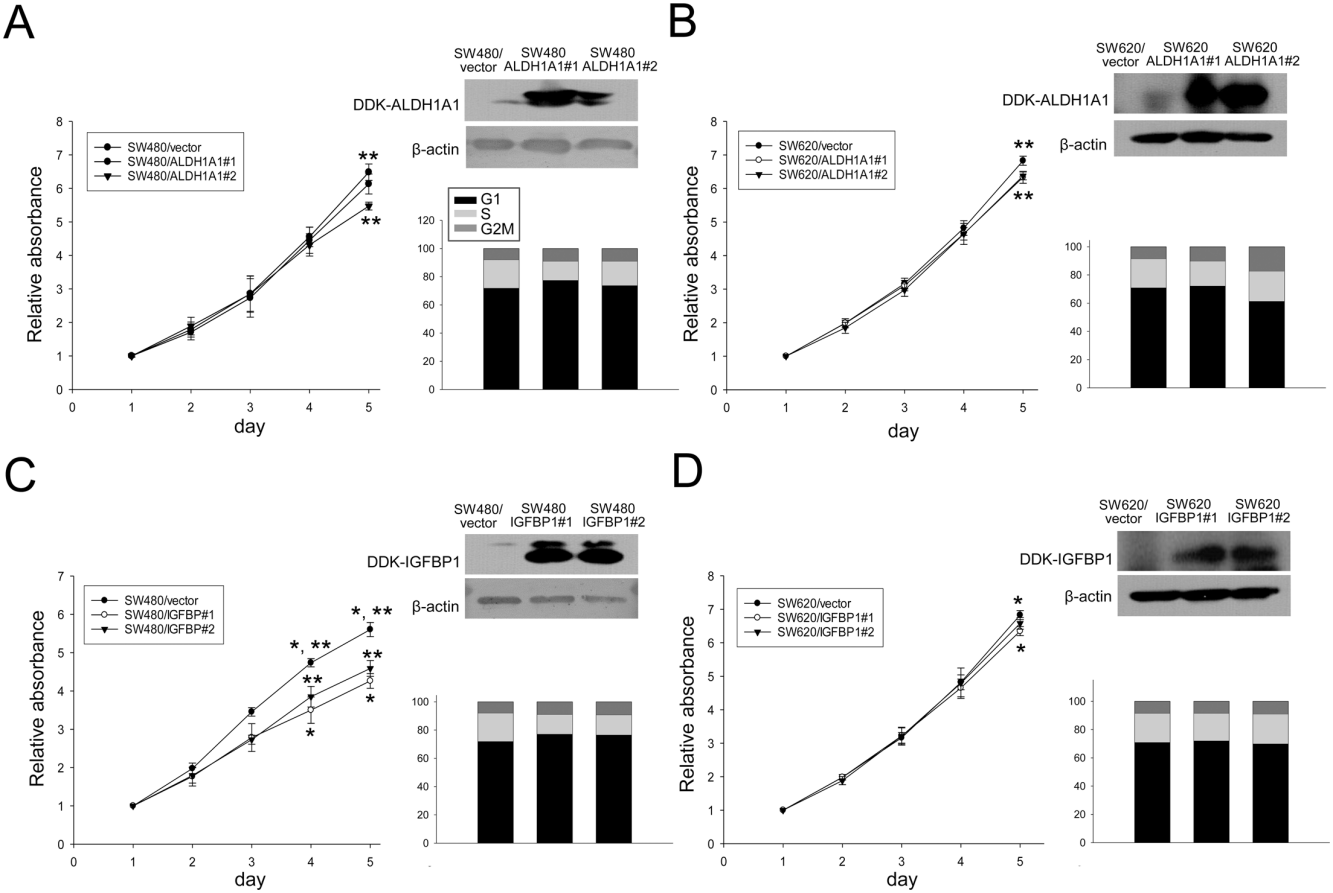
Proliferation and cell cycle assays using *ALDH1A-* and *IGFBP1-*transfected cells. ALDH1A1and IGFBP1 specifically decrease CRC cell proliferation (left column) and accumulation of cells in the S and G2/M phases (right column) in SW480 cells (A and C) and SW620 cells (B and D). The cell proliferation rates were measured using the CCK8 cell proliferation assay and expressed as daily proliferation rate. Cell-cycle phases were measured on a flow cytometer with propidium iodide staining. *p < 0.05 between vector and clone #1, and *p* < 0.05 **between vector and clone #2, respectively.

ALDH1A1- and IGFBP1-overexpressing CRC cells were generated by cDNA transfection to acquire at least two clones and were subsequently used to measure the effects of these proteins on the proliferation of CRC cells (Fig 3). Overexpression of *ALDH1A1* and *IGFBP1* dramatically reduced the proliferation rate of SW480 cells in a time-dependent manner, which became evident after between 4 and 5 days (*p* < 0.05). Similarly, ALDH1A1-overexpressing SW620 cells showed a reduced proliferation rate on day 5. The effects of ALDH1A1 and IGFBP1 on the cell cycle distribution were examined. Consistent with the results of proliferation assays, ALDH1A1- and IGFBP1-overexpressing SW480 cells showed decreased accumulation of cells in the S and G2/M phases compared with untreated SW480 cells, whereas the differences were not significant in SW620 cells.

### ALDH1A1 and IGFBP1 inhibit invasion and migration of CRC cells

**Fig 4:**
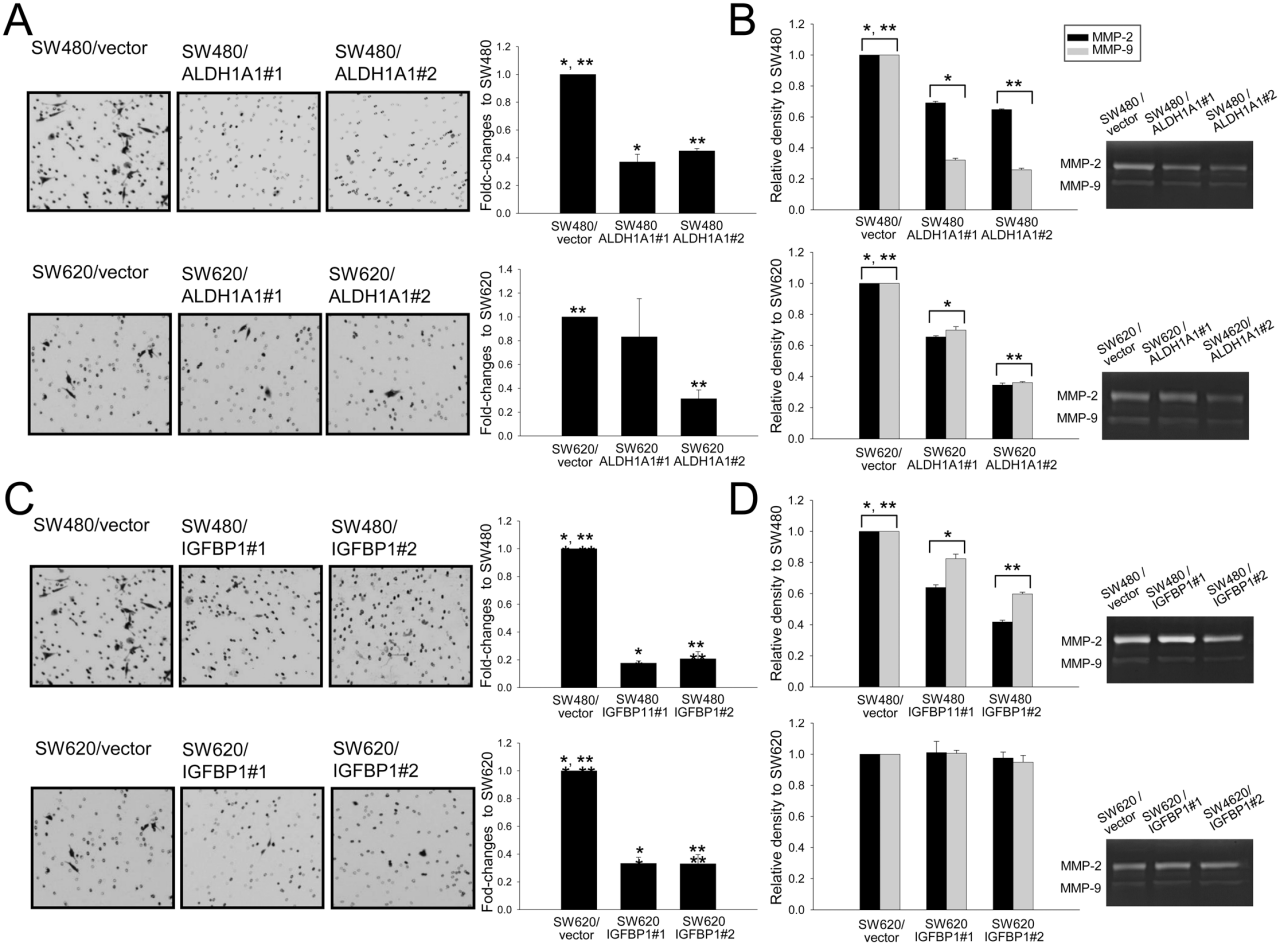
Invasion and migration assays using *ALDH1A-* and *IGFBP1-*transfected cells. Invasiveness was measured by the number of CRC cells invading into the lower chamber in the well (A and C) and by the gelatinolytic activity of MMP-2 and MMP-9 (B and D). All treated cells showed significantly reduced invasiveness in these assays except IGFBP1-overexpressing SW620 cells on gelatin zymography. *p < 0.05 *between vector and clone #1 and **p < 0.05 between vector and clone #2, respectively.

Invasiveness was measured using the number of CRC cells invading the Transwell chamber and expressed as the fold change between overexpressing and control cells (Fig 4A). IGFBP1-overexpressing SW480 and SW620 cells showed a significant 3.0–5.7-fold lower number of invading cells than control cells (*p* < 0.05). ALDH1A1-overexpressing SW480 cells similarly showed a >2-fold reduction in invasiveness (*p* < 0.05), and ALDH1A1-overexpressing SW620 cells showed a tendency toward reduced invasiveness compared with control cells. The effect of ALDH1A1 and IGFBP1 on MMP activity was measured by gelatin zymography (Fig 4B), which showed that the relative activities of MMP-2 and MMP-9 in ALDH1A1-overexpressing SW480 and SW620 clones were significantly reduced by 1.4–3.9-fold compared with their respective control cells (*p* < 0.05). The relative densities of MMP-2 and MMP-9 activity bands were significantly reduced in IGFBP1-overexpressing SW480 cells, but not in the SW620 cell line. Otherwise, both focal adhesion kinase (FAK) and phospho-FAK (p-FAK) were underexpressed in IGFBP1-overexpressing SW480 cells, and the reverse tendency was seen in SW620 cells (S3A Fig).

### Expression of molecules associated with epithelial-mesenchymal transition and CRC stem cells

**Fig 5:**
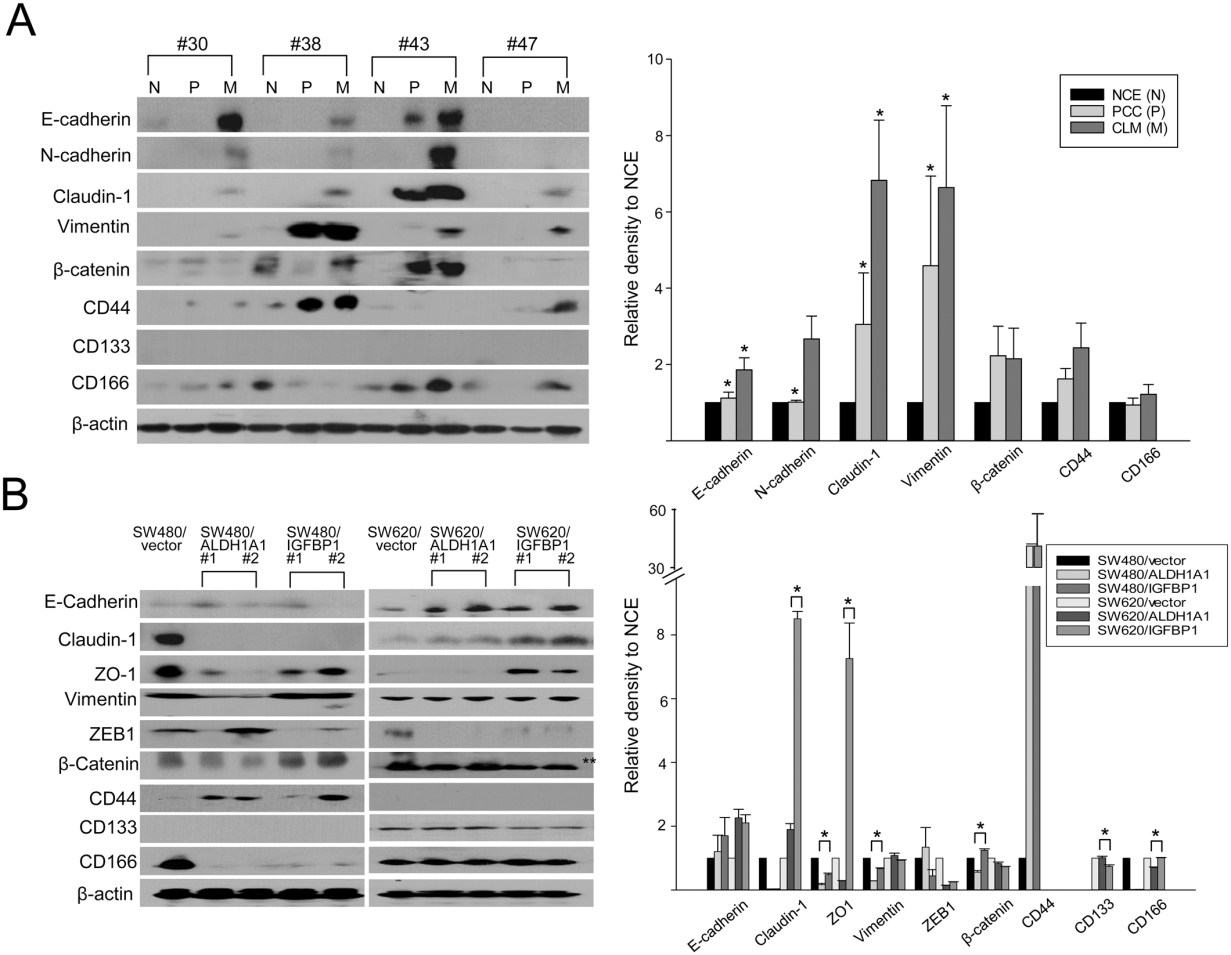
Expressions with relative densities of epithelial-mesenchymal transition/CRC stem cell (EMT/CSC)-related molecules. CRC patients with CLM (A), vector, ALDH1A1- and IGFBP1-overexpressing CRC cells (B). *p < 0.05 between PCC and CLM or between vector and ALDH1A1- or IGFBP1-overexpressing clones. NCE (N), normal colic epithelium; PCC (P), primary colorectal cancer; CLM (M), colorectal cancer liver metastasis.

We evaluated the expression of 12 epithelial-mesenchymal transition (EMT)/CSC-related molecules using available tissue samples, vector-treated cells, and ALDH1A1- and IGFBP1-overexpressing cells (Fig 5). The expression levels of E-cadherin, N-cadherin, claudin-1, and vimentin were significantly higher in CLM than in PCC tissues (*p* < 0.05), whereas bands corresponding to snail, slug, ZEB1, and CD133 were not detected in any of the matched tissue samples. Expressions of β-catenin, vimentin, and ZO-1 were significantly greater or maintained in IGFBP1-overexpressing SW480 cells than in ALDH1A1-overexpressing and control SW480 cells, respectively (*p* < 0.05). Otherwise, expression of the four markers including claudin-1, ZO-1, and CD166 were significantly greater or maintained in IGFBP1-overexpressing cells than in their ALDH1A1-overexpressing and control SW620 cells, respectively (*p* < 0.05). Expression of CD44 was not detected in SW620 cells, whereas CD133 expression was not identified in SW480 cells.

### IGFBP1 promotes liver metastasis in SW480-cell transplant mice

**Fig 6:**
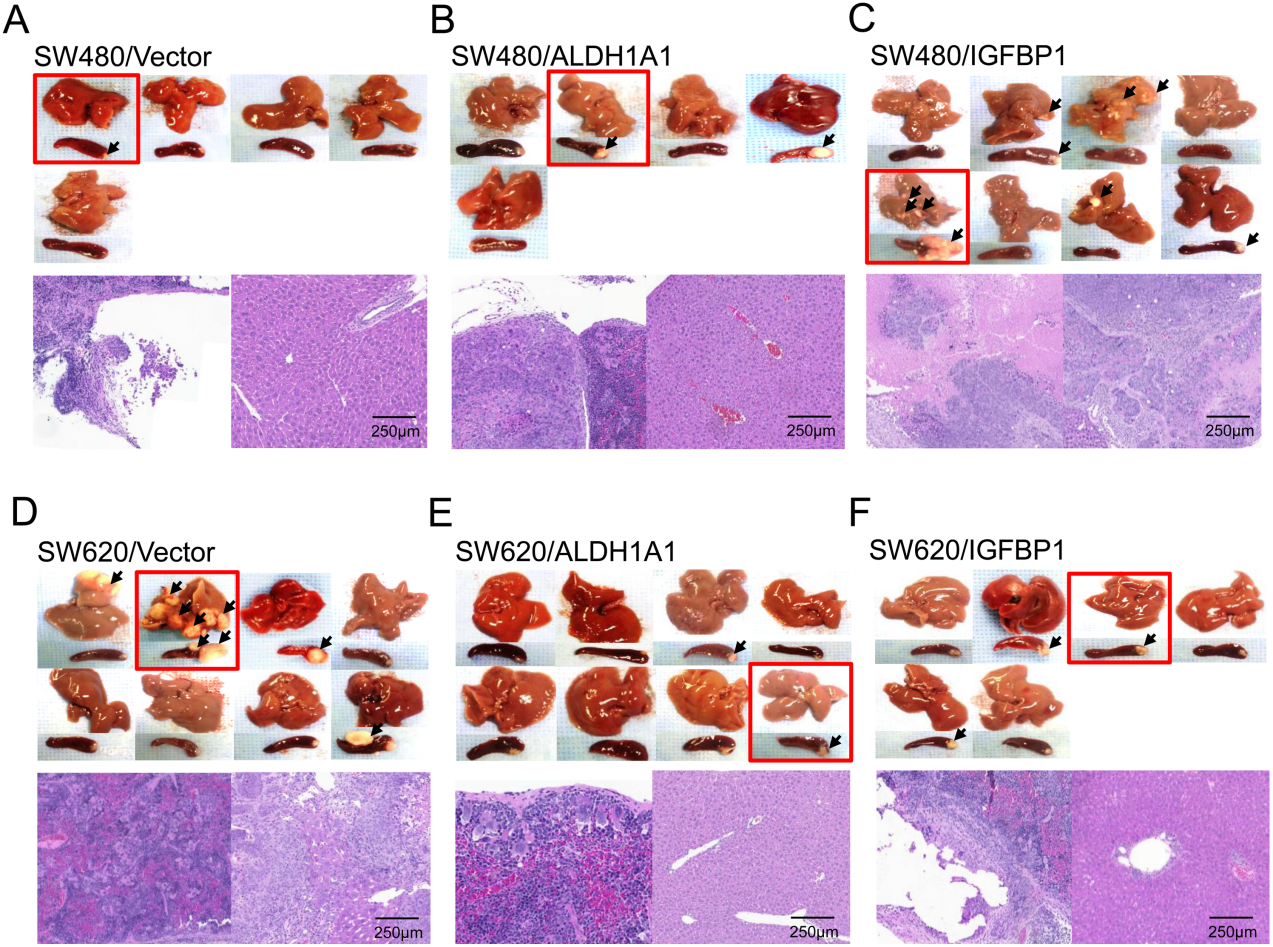
Primary tumors at the spleen (transplant site) and liver metastases. Arrows indicated tumors (the spleen and liver were set at the upper and lower parts, respectively) and histological views (H & E, ×100; photomicrographs of the spleen and liver were set at the left and right sides, respectively, from red squared specimens). CLM, colorectal cancer liver metastasis. The mice with CLM was in order (right and downward) with long diameter: A, no CLM; B, no CLM; C, #2 (2.7 mm) #3 (2–5.3 mm) #5 (4.4–6.6 mm) #7 (4.4 mm); D, #1 (16.0 mm) #2 (2.2–8.0 mm); E, no CLM; F, no CLM.

After the exclusion of 8 mice that died of tumor-unrelated causes, primary spleen tumors (transplant site) and liver metastases were identified grossly and histologically in 40 mice (Fig 6). Primary tumors were successfully implanted and grew by 20–50% in all groups. Liver metastasis exclusively occurred in mice implanted with IGFBP1-overexpressing SW480 cells, whereas it was not identified in control mice or mice implanted with ALDH1A1-overexpressing SW480 cells (4/8 mice *vs*. 0/10 mice, *p* = 0.023). On the other hand, liver metastasis occurred in mice treated with control SW620 cells (2/8 mice), whereas it was not identified in mice treated with ALDH1A1- or IGFBP1-overexpressing SW620 cells. The long diameter of the CLMs was in the range of 2.0–16 mm. Irrespective of whether SW480 or SW620 cells were used, vector-transfected mice did not express IGFBP1 in the spleen or liver, whereas it was overexpressed in the spleen and liver of *IGFBP1*-transfected SW480 xenografted mice with CLM (S1B Fig). Otherwise, ALDH1A1 was over-expressed in the liver regardless of cell lines. We further examined β-catenin and its target molecules such as c-Myc and survivin in our xenografts of IGFBP1- and ALDH1A1-overexpressing SW480 cells. The former xenograft with CLM showed concurrent overexpression of β-catenin and c-Myc, and the latter one without CLM also expressed them except for c-Myc in the spleen (S3B Fig).

## Discussion

Various gene clusters can be efficiently harvested from a single RNA-Seq data set according to the specific aim and bioinformatics tools used. We initially selected 998 genes showing ≥2-fold differential expression in PCC and CLM tissues. The reproducibility of the selected genes was re-examined according to their RNA-Seq score using quantitative RT-PCR and western blotting in a second cohort. Core genes selected according to differential expression using RNA-Seq need to be validated in a second cohort using strict criteria, which include their reproducibility and few post-transcriptional changes [18]. We excluded liver-specific genes because their expression tends to be labile and is affected by metabolic changes and toxic damage in hepatocytes, independently of the presence of CLM. We further narrowed down the selection to nine genes on the basis of functional association with the metastatic process of CRC. Two genes, *ALDH1A1* and *IGFBP1*, were finally selected to examine CLM-related biological interactions because they were remarkably upregulated in CLM tissues and concurrently showed few post-transcriptional and post-translational changes. Regarding the specificity of these genes in metastatic tumors, the expression levels of ALDH1A1 and IGFBP1 were increased by 15.6% and 6.3%, respectively, compared with normal liver tissues, considering a contamination rate of <3%. However, the paracrine effect of ALDH1A1 and IGFBP1 from contaminated liver tissues was considered, even if it was weak.

ALDH1A1 mRNA and protein expressions were significantly greater in CLM than PCC tissues of our patients with synchronous CLM. One IHC study reported that a lower ratio of the ALDH1A1 level in adjacent mucosa to that of the tumor tissue (RA/C <1) positively correlated with tumor invasion and metastasis capabilities [19]. Unexpectedly, ALDH1A1 overexpression reduced the proliferation and invasion of CRC cells and decreased MMP-2 and MMP-9 activity in our study. MMP-2 and MMP-9 play important roles in the degradation of the extracellular matrix and basement membrane, promoting tumor invasion and metastasis [20]. These findings suggest that ALDH1A1 overexpression in CLM may act as a metastasis inhibitor rather than a metastasis inducer. If we consider ALDH activity to be a hallmark of CSCs, possessing indefinite proliferation and metastatic potential [21], overexpression in CLM tissue hardly appears to explain the reduced proliferation and invasion of ALDH1A1-overexpressing cells. ALDH1A1 may be either a cause or consequence of CLM and further work is required to determine its exact role.

Despite the lack of sound evidence supporting the stimulation of tumor growth and migration by IGFBP1, the mRNA and protein levels of IGFBP1 were significantly higher in CLM than PCC tissues in our study. In contrast, IGFBP1-overexpressing SW480 cells showed lower rates of proliferation and invasion than untreated cells in our cell assays. IGFBP1 has independent inhibitory effects on cancer cell growth and metastasis in preclinical studies, both directly and through local modulation of other components of the IGF axis [4,22]. The other observational study reported that higher levels of plasma C-peptide and lower levels of plasma IGFBP-1 at prediagnosis were associated with increased mortality in patients with non-metastatic CRC [5].

EMT/CSC is a critical early event in CRC invasion and metastasis, and it is characterized by the presence of specific markers for each phenotype [23]. CD133, CD44, CD166, ALDH1A1, and Lgr5 are CRC stem cell markers [6]. EMT triggers reversion to a CSC-like phenotype. In the four CRC patients with CLM included in the current study, the expressions of E-cadherin, N-cadherin, claudin-1, and vimentin were significantly higher in CLM than NCE and PCC tissues, whereas snail, slug, ZEB1, and CD133 showed no detectable expression in CLM tissues. Additionally, the so-called “cadherin switch”, which consists of N-cadherin overexpression and reduced E-cadherin expression and is an integral component of the EMT [24], was not detected in CLM tissues or CRC cells. The markers overexpressed varied depending on the genes transfected and the cells, even if their clones were derived from the same patient (SW480 and SW620). Taken together, all EMT/CSC marker expressions differed in the respective clone and thereby appeared to play diverse roles during CRC progression. CD44 expression was significantly reduced in SW620 cells, whereas CD133 expression was reduced in SW480 cells. Knockdown of CD44 resulted in limited colony formation and reduced tumor formation in xenografts, strongly indicating a functional role of CD44 in CRC tumorigenesis [25]. CD133 has been considered a marker of colon CSCs and an indicator of aggressiveness and metastasis [20]. Downregulation of these CSC markers might affect the proliferation and invasiveness of transfected SW480 and SW620 cells.

Liver metastasis exclusively occurred in mice with intrasplenic injection of IGFBP1-overexpressing SW480 cells, which might be partly explained by the persistent expression of β-catenin, vimentin, and ZO-1 in these mice. β-catenin and c-Myc were concurrently overexpressed in xenograft tissues from mice injected with IGFBP1-overexpressing SW480 cells. Deregulation of Wnt–β-catenin signaling facilitates constitutive renewal and aberrant expansion of CSCs, in collaboration with downstream molecules such as c-Myc and survivin [26–28]. On the other hand, the EMT-related vimentin is a marker used to detect epithelial transition to the fibroblastoid phenotype, and ZO-1 re-expression was identified in the CLM tissues of CRC [29,30]. FAK with activated p-FAK is overexpressed in invasive and metastatic colon cancer [31]. In the current study, FAK and p-FAK were underexpressed in IGFBP1-overexpressing SW480 cells; these cells concurrently showed reduced proliferation and invasion. Our contradictory findings between the *in vivo* and *in vitro* experiments may indicate the potential existence of an unidentified behavior or alternative pathway essential for the survival of tumor cells in the CLM tumor microenvironment. At this point, we may also consider that the animal model provides more accurate evidence than in the *in vitro* experiments by mirroring the pathological changes in human.

In our intrasplenic xenograft model, CLM was detected in 25% of mice transplanted with untransfected SW620 cells, whereas no CLM was found in IGFBP1-overexpressing SW620 cells. This cell line is known as a metastatic and chemo-resistant one cloned from metastatic lymph nodes of the same patient who incurred primary SW480 [32]. Because IGFBP1 expression was not detected either in the spleen and liver tissues of vector-transfected or I*GFBP1*-transfected SW480 cells and SW620 cells without CLM, IGFBP1 could be inactivated in the normal liver with unidentified mechanism, in addition to a possible lack of endogenous IGFBP1 in these cells. FAK and p-FAK were over-expressed in IGFBP1-overexpressing SW620 cells, contrarily to a reduced proliferation and invasion in the *in vitro* cell assays and no CLM in the xenografts implanted with these cells. On the other hand, downregulation of adhesion molecules such as E-cadherin decreases epithelial cell-to-cell adhesion, leading to the acquisition of a spindle-shaped, highly motile, fibroblast-like phenotype during EMT [33]. Loss of the “cadherin switch” in our study partly explains the suppression of liver metastasis observed in ALDH1A1- and IGFBP1-overexpressing SW620 cells.

In our current study, ALDH1A1, which showed higher expression in CLM than PCC tissue, did not consistently act as a metastasis-promoting gene that was correlated with poor clinical outcome, as previously reported [21]. Furthermore, IGFBP1 may have a dual function, playing both positive and negative roles in the progression and metastasis of CRC. Although these findings are based on the strict criteria used for gene selection and biological validation, there are some limitations to the strength of the conclusions that can be reached in the current study. Important genes associated with CLM may be missed because of type I errors, which are associated with a limited sample size. The possibility of such an error in the *in vivo* experiments cannot be excluded due to the small number of mice. Nevertheless, our results suggest the existence of unidentified behaviors of ALDH1A1 and IGFBP1 that may facilitate the reassessment of their potential value as CLM modulators and as biomarkers or therapeutic targets in future pathway-related and clinical validation studies.

## Supporting Information

S1 Fig The mRNA and protein expressions of ALDH1A1 and IGFBP1 in various cell lines and xenografts. (A) Relative mRNA expressions were shown in two normal colonic epithelial cell lines and ten CRC cell lines. (B) Protein expressions in the spleen and liver of mice implanted with vector-, ALDH1A1-, and IGFBP1*-*overexpressing SW480 and SW620 cells.
(TIF)

S2 Fig Randomly selected samples examined for contamination by normal liver tissues. Left column, H & E staining; middle column, immunohistochemistry (IHC) using hepatocyte-specific Heppar-1; right column, bile-duct-cell-specific IHC using CK-7. None (A) or a few (B) positively stained cells were identified.
(TIF)

S3 Fig Expressions of focal adhesion kinase (FAK) and β–catenin with its target molecules. FAK and phospho-FAK expression in IGFBP1-overexpressing SW480 cells and SW620 cells (A) and expressions of β–catenin with its target molecules in SW480 xenografts (B).
(TIF)

S1 Table Demographic and biological features in the two groups of initial RNA sequencing and identifying candidate gene expressions, respectively. (PDF)

S2 Table The 998 genes differentially expressed with ≥2-fold changes between PCC and CLM by RNA-Seq based on a GLM likelihood ratio test. (PDF)

S3 Table The 97 genes consistently upregulated in >50% of CRC patients examined. (PDF)

S4 Table Primers and conditions for real time RT-PCR in the 9 selected genes. (PDF)
