# Supplementary figures and images for: Complex Behavior of ALDH1A1 and IGFBP1 in Liver Metastasis from a Colorectal Cancer
Source: PLoS One. 2016 May 6;11(5):e0155160. doi: 10.1371/journal.pone.0155160 (PMC4859559; doi:10.1371/journal.pone.0155160)

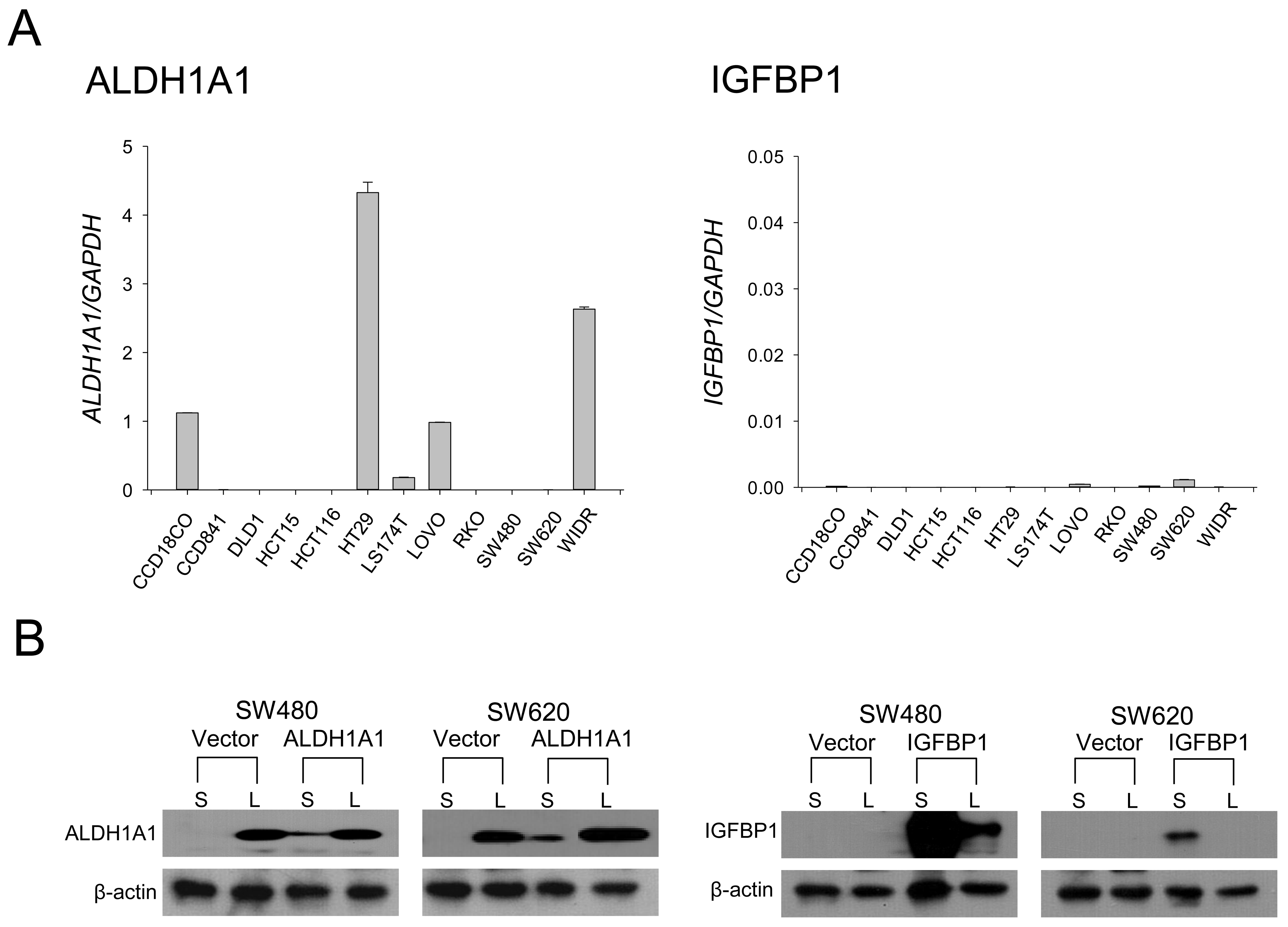

Supplement: S1 Fig — (A) Relative mRNA expressions were shown in two normal colonic epithelial cell lines and ten CRC cell lines. (B) Protein expressions in the spleen and liver of mice implanted with vector-, ALDH1A1-, and IGFBP1-overexpressing SW480 and SW620 cells. (TIF) [file pone.0155160.s001.tif]

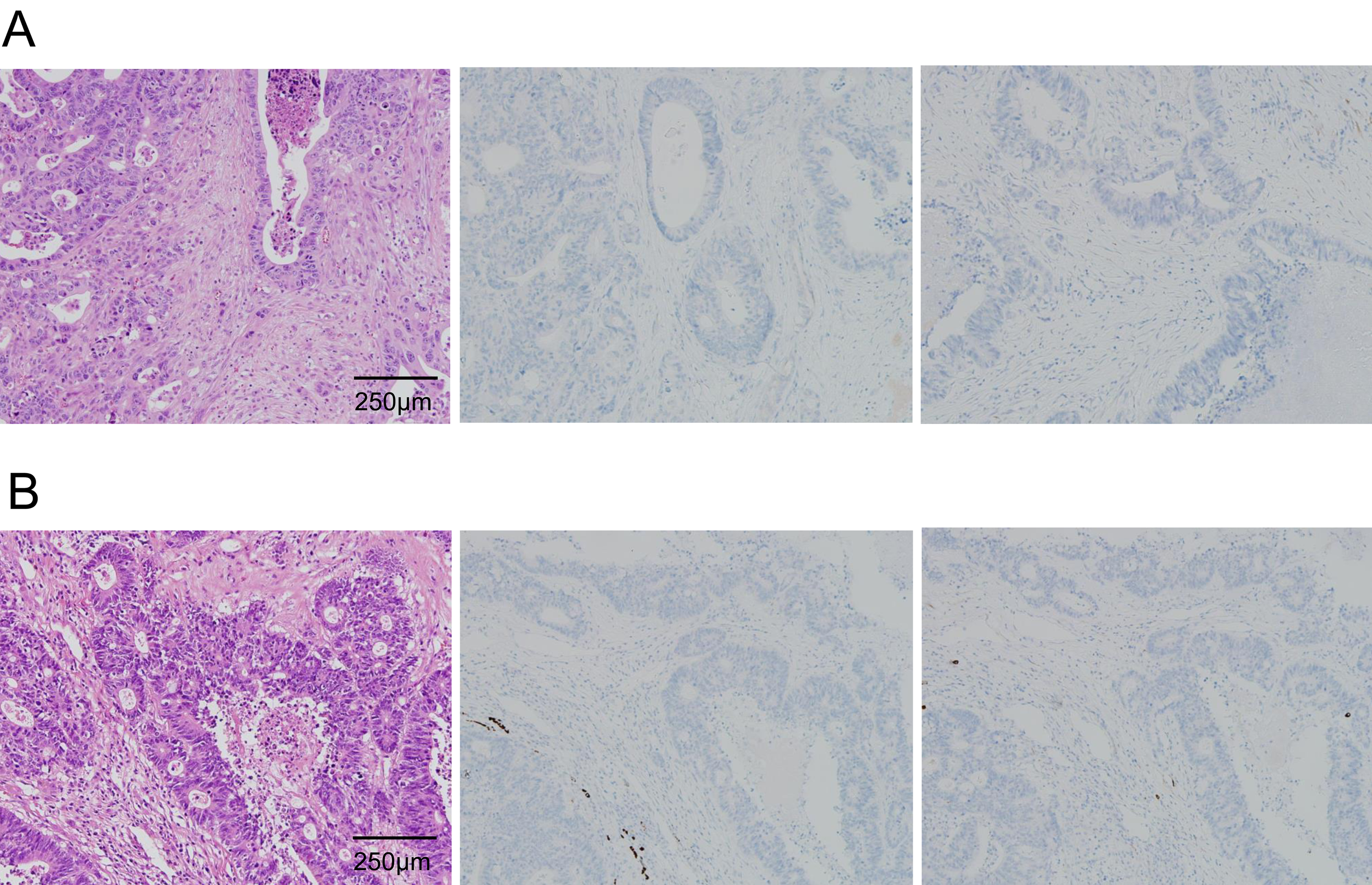

Supplement: S2 Fig — Left column, H & E staining; middle column, immunohistochemistry (IHC) using hepatocyte-specific Heppar-1; right column, bile-duct-cell-specific IHC using CK-7. None (A) or a few (B) positively stained cells were identified. (TIF) [file pone.0155160.s002.tif]

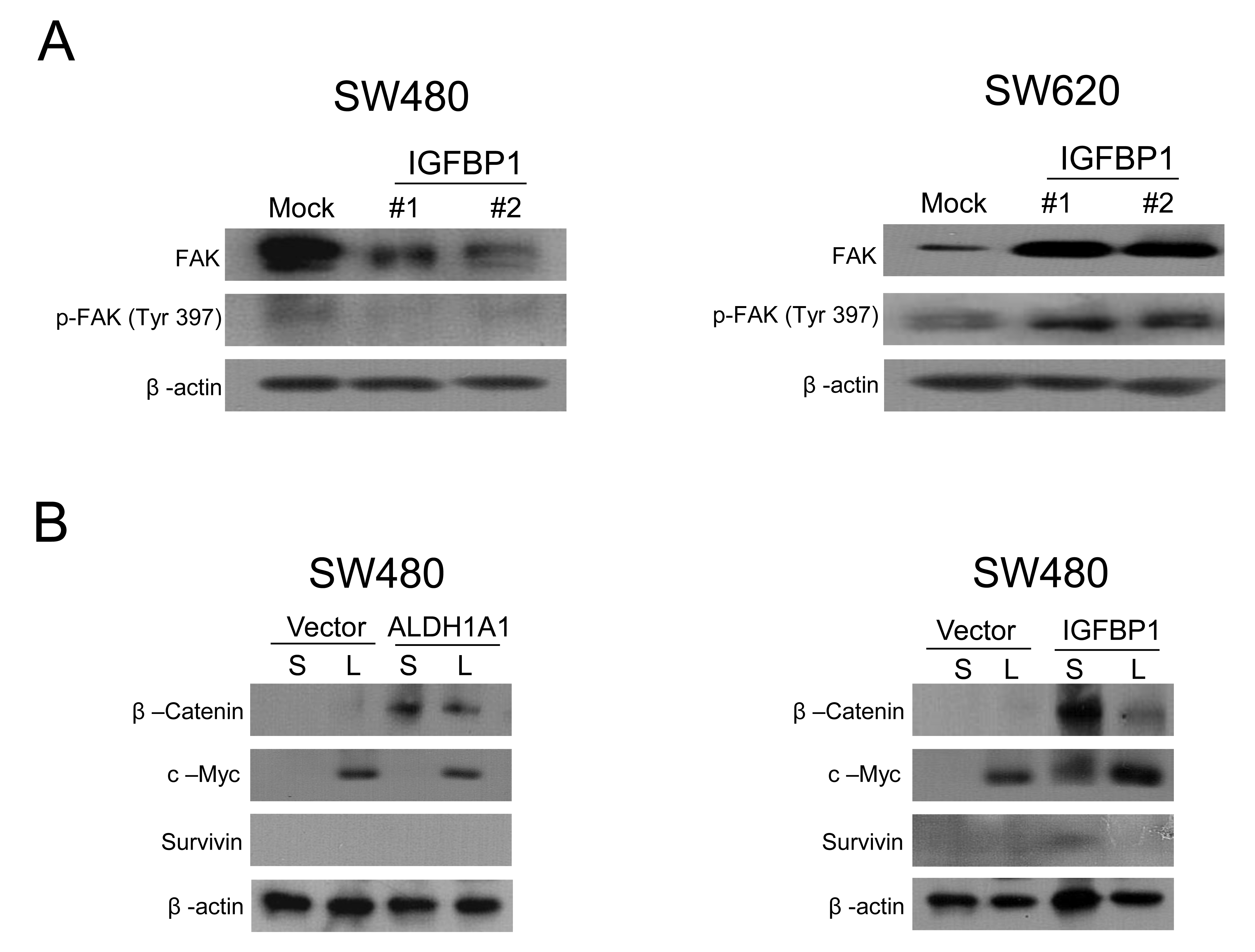

Supplement: S3 Fig — FAK and phospho-FAK expression in IGFBP1-overexpressing SW480 cells and SW620 cells (A) and expressions of β–catenin with its target molecules in SW480 xenografts (B). (TIF) [file pone.0155160.s003.tif]
