# Supplementary material for: Complex Behavior of ALDH1A1 and IGFBP1 in Liver Metastasis from a Colorectal Cancer
Source: PLoS One. 2016 May 6;11(5):e0155160. doi: 10.1371/journal.pone.0155160 (PMC4859559; doi:10.1371/journal.pone.0155160)
Supplement: S1 Table — (PDF) [file pone.0155160.s004.pdf]

S1 Table. Demographic and biological features in the two groups of initial RNA sequencing and identifying candidate gene expressions, respectively

Parameters, number of patients or mean ± SD	Group for initial RNA sequencing, n = 18	Group for candidate gene expressions, n = 10	*p*-value[a]
Sex, male/female	4/14	1/9	0.626
Age, years	60 ± 10	58 ± 8	0.567
Primary tumor site, colon/rectum	10/8	7/3	0.689
CLM, single/multiple	11/7	4/6	0.433
Level of serum CEA, ng/ml	70.6 ± 163.2	47 ± 99	0.677
Metastatic lymph node+	12	6	1
Lymphovascular invasion+	8	6	0.695
Perineural invasion+	7	4	1
Differentiation of PCC, WD/MD/PD	0/18/0	9/1	0.357
MSI of PCC, MSS/MSI-L/MSI-H	17/0/1	10/0/0	1
p53 expression of PCC[b], 0-1/2-4	7/11	4/5	1
ALDH1A1 mRNA overexpression[b]	14	9	0.626
IGFBP1 mRNA overexpression[b]	16	9	1

SD, standard deviation; PCC, primary colorectal cancer; CLM, colorectal liver metastasis; CEA, carcinoembryonic antigen; WD/MD/PD, well-/moderately-/poorly-differentiated; MSI, microsatellite instability; MSS, microsatellite stable; MSI-L, MSI with low frequency; MSI-H, MSI with high frequency; ISR, intersphincteric resection; pStage, pathological AJCC stage; n.a., not applicable. Bold prints, $p<0.05$.

[a]All parameters were compared using Fisher's exact test with two-sided verification or Pearson's χ^2 test and an unpaired Student's *t*-test.

[b]Nuclear expression of p53: 0, no expression; 1, 0–≤25%; 2, >25–≤50%; 3, >–≤75%; 4, >75%.

[c]Fold-changes of mRNA expression compared to that of normal colic epithelium: if greater in CLM than in PCC.
