# Supplementary material for: Complex Behavior of ALDH1A1 and IGFBP1 in Liver Metastasis from a Colorectal Cancer
Source: PLoS One. 2016 May 6;11(5):e0155160. doi: 10.1371/journal.pone.0155160 (PMC4859559; doi:10.1371/journal.pone.0155160)
Supplement: S2 Table — (PDF) [file pone.0155160.s005.pdf]

**Genes**	**Average fold-count (log_2)**	***p* -value**	**Patient count_2-fold.upreg**
TTR	7.13246658	3.89016E-80	15
HULC	7.110014914	4.71869E-72	16
COLEC11	4.693084634	9.434E-52	16
RGN	4.590994059	1.49992E-42	15
HSD11B1	4.223696746	1.24953E-39	15
UGT2B7	3.760798145	1.32292E-33	15
PI3	-2.28452465	3.65365E-13	3
GREM1	-2.440802166	4.40987E-18	1
ACTG2	-3.012259522	3.34538E-26	0
FOXF1-AS1	-3.965674969	1.25868E-41	0
SFRP2	-5.391409568	1.80624E-50	2
MBL2	9.631673707	1.0133E-102	15
CFHR2	9.233561013	1.3846E-103	15
CYP4A11	9.087745669	1.3385E-109	15
ACSM2B	8.867224074	7.05921E-98	15
APOF	8.585492489	6.7431E-100	15
IGFBP1	8.526850893	1.0884E-110	15
ACSM2A	8.40099595	1.89435E-88	15
CFHR1	7.901049177	1.71305E-90	15
SLC25A47	7.721720613	4.56813E-89	15
CYP8B1	7.683404706	7.03741E-87	15
FTCD	7.585255957	2.30162E-83	15
CFHR3	7.453849414	8.201E-86	15
APOC1P1	7.411447954	8.63625E-69	15
HGFAC	7.400657726	1.30703E-79	15
C19orf80	6.923822306	3.76057E-73	15
CCL16	6.360925556	5.73516E-63	15
HSD17B13	6.344763476	4.72965E-71	15
SLC38A3	6.033892733	1.49019E-63	15
AMDHD1	4.405429395	3.1682E-43	15
MARCO	3.696925914	8.1528E-34	15
APOE	2.847386648	9.64467E-23	15
SERPING1	2.303314031	3.60557E-15	13
PLIN2	2.051568417	1.16304E-14	14
ACSL1	1.876769805	9.40365E-13	15
TFF1	-1.102501132	0.000142329	4
CTSK	-2.124807007	1.97971E-15	1
MMP2	-2.38120298	1.85926E-18	1
CCL11	-3.817980631	6.64106E-34	0
DES	-3.910860212	1.852E-35	0
PRODH2	10.45299796	7.43614E-95	14
CFHR5	10.12060992	6.9752E-110	14
FCN2	9.372428863	3.11972E-86	14
APOA5	8.900869772	7.8448E-100	14
UGT1A4	8.839752501	4.91597E-88	14
CD5L	8.446057729	9.33554E-92	14
CPN2	8.135863979	1.0968E-90	14
CFHR4	7.752500118	8.55989E-71	14
C5orf27	6.551056841	1.1035E-65	14
GCKR	6.26874127	1.18837E-69	14
INHBE	5.384028367	1.026E-54	14
ABCG5	4.587969406	1.07639E-40	14
OGDHL	4.104351312	3.27782E-41	14
TTC36	3.857967212	6.98573E-32	14
ASPDH	3.82378906	9.87635E-33	14
ABCB4	3.422083126	5.09014E-30	14
NPW	3.027450759	1.35903E-22	13
CBS	2.84257187	7.45393E-23	14

VNN1	2.823145519	7.93878E-22	13
F10	2.815195593	7.42456E-23	14
CLU	2.337354585	2.32858E-13	11
CFB	2.235136199	4.94956E-11	13
ATF5	1.945399486	6.87562E-14	14
GOLT1A	1.872029361	4.8212E-12	13
ALDH1L1	1.868237272	2.30653E-08	11
MT1L	1.633439888	3.39053E-08	12
RBP1	1.238128284	7.79009E-06	11
MUC5B	-1.42511005	2.5673E-05	5
FOXF1	-2.205316487	1.7775E-16	0
SLCO2A1	-2.29182642	3.5767E-15	2
CPA3	-2.38784122	2.07523E-17	1
SPARCL1	-2.556764702	1.29158E-19	0
DMBT1	-2.862781019	1.04333E-16	2
SLC17A2	10.18921453	7.80512E-91	13
CYP4A22	10.00851757	7.27596E-91	13
UGT3A1	9.524586197	3.72264E-87	13
CYP1A2	8.575277437	5.47536E-85	13
ACOT12	8.442196923	1.66636E-78	13
RTP3	7.566177574	5.68153E-58	13
ACMSD	6.440506414	1.68808E-57	13
THRSP	6.290040867	8.44952E-59	13
PRG4	5.548321676	3.47953E-51	13
UGT2B15	5.06604696	3.38465E-32	12
AGMO	3.640570984	6.69632E-31	13
CDH2	2.935506558	2.26191E-23	13
ALPL	2.661990792	5.28926E-20	13
C2	2.110940655	8.36275E-05	11
TTPA	1.993738301	1.74696E-13	13
CYP4F3	1.928175826	4.51697E-13	12
FBP1	1.487935031	1.61427E-08	12
LOC100505989	-1.372406122	5.46783E-06	2
F3	-1.500516158	2.28422E-08	2
LCN2	-1.926043736	2.99832E-10	2
APOD	-1.949501952	2.54458E-11	1
CLC	-2.19736349	7.92404E-13	1
TSPAN11	-2.94515679	9.08929E-26	0
D4S234E	-3.120220543	1.98486E-24	1
TRPA1	-4.511627002	4.07686E-45	1
CLEC1B	7.173721755	5.91824E-58	12
LOC100507203	6.458996198	1.08335E-61	12
LOC255167	5.974686108	1.02961E-58	12
ABCG8	4.872092774	1.28043E-41	12
SHBG	4.871236821	3.99741E-40	12
PPP1R1A	4.156411501	8.94094E-32	12
SLC6A1	3.206191633	5.45686E-26	12
SLC6A12	2.946365793	1.05384E-22	12
FNDC4	2.381536454	3.98395E-16	12
GLTPD2	2.164768905	5.56461E-15	12
PC	1.871472032	3.35338E-12	12
IGFBP2	1.726096457	2.54111E-10	11
MT1A	1.717138871	3.60048E-09	11
RBP5	1.697234945	1.06294E-09	10
KYNU	1.58628401	2.00968E-09	11
BNIP3	1.507264811	1.13815E-08	11
CD163	1.233002118	3.61917E-06	10
C10orf10	1.145664833	3.03527E-05	9
ZG16B	-1.226429628	5.63156E-06	1

VWA5A	-1.321666517	2.20594E-07	1
PDGFRA	-1.379187851	2.36292E-07	2
DKK3	-1.517611555	6.65736E-09	1
CNN1	-1.793207646	1.38652E-10	1
PLA2G2A	-1.913905344	1.67354E-10	1
LEFTY1	-2.027648798	9.56352E-13	1
DUOX2	-2.133008515	3.80721E-12	2
PCDH18	-2.241747185	2.26068E-17	0
VWF	-2.479302873	7.59327E-20	0
CLMP	-3.087326955	4.87731E-25	0
PLGLA	12.44811825	2.87609E-59	11
GDF2	9.83827282	1.50252E-83	11
APOA4	7.977596211	4.35189E-65	11
UGT1A3	5.154384523	1.01416E-34	11
SARDH	3.413107732	3.51482E-30	11
MASP1	3.01647454	2.26414E-19	10
IGFALS	2.913924297	3.52273E-19	10
PLIN5	2.538876072	9.79766E-18	10
SLC7A2	2.296812184	7.44442E-15	11
CYP1B1	2.179163689	2.4004E-13	11
LEPR	2.152320326	2.20005E-14	11
NGFR	1.876611252	1.46195E-10	10
ADAMTSL2	1.832907568	1.30556E-11	10
ALDH1A1	1.738217681	3.007E-10	11
ENPEP	1.676395882	4.21645E-10	11
CFP	1.621564866	7.62346E-09	10
RND1	1.571333528	2.5221E-09	11
CSTA	1.510206665	1.56921E-08	10
G0S2	1.495680362	4.93453E-08	10
EFHD1	1.435270626	3.05238E-07	9
INMT	1.317285846	5.24458E-07	10
PROS1	1.299826035	7.05684E-07	10
TREM2	1.271422861	4.13962E-06	10
HHEX	1.237190927	3.81587E-06	9
CYB5A	1.233442981	9.72097E-07	11
C1S	1.230094885	1.24835E-05	9
C10orf125	1.173270276	7.61711E-06	10
CD14	1.124617057	2.0217E-05	10
EPHX1	1.123020046	1.47549E-05	10
CXCL9	1.056135771	0.000202661	9
SLC43A3	1.049810241	6.2967E-05	9
PHGDH	1.019517046	0.000145982	10
ALDH6A1	1.002765585	0.000239323	9
APCDD1	-1.177774584	1.6264E-05	1
RSPO3	-1.247813462	2.55052E-05	1
ENPP3	-1.256360877	1.08022E-05	2
SOD3	-1.285627841	1.37983E-06	1
LUM	-1.310824437	6.06201E-07	1
CCL13	-1.482076791	4.15102E-06	2
MRVI1	-1.501834424	9.44259E-09	0
CAV1	-1.546532553	3.13812E-09	0
PLAT	-1.553943911	3.57691E-09	0
ITIH5	-1.661583509	1.54338E-09	1
NOS2	-1.767273411	1.35512E-10	1
PLN	-1.998680626	1.89795E-11	1
DARC	-3.17471426	6.87239E-23	0
FAM99A	10.33742636	4.63982E-80	10
SRD5A2	9.93653373	1.65613E-78	10
FGF21	8.560656534	8.70428E-60	10

SLC22A10	7.928908021	4.53246E-67	10
ART4	4.248424919	1.11048E-35	10
SLC47A1	2.803543799	4.78745E-22	10
PLA2G12B	2.438353935	5.75537E-14	9
GLDC	2.381163355	1.02709E-14	9
ACSS3	2.223845893	3.17728E-14	10
CETP	1.952559611	3.85585E-12	10
FAM20A	1.934924446	1.20947E-11	9
CYP27A1	1.85319621	1.08477E-11	10
LYNX1	1.745540842	3.54971E-09	8
LCAT	1.728658535	4.31688E-10	10
CYP2D7P1	1.59493288	2.16458E-09	10
ERRFI1	1.41189985	2.25357E-08	10
GPD1	1.394982262	1.28142E-07	10
FABP3	1.372755342	4.00556E-07	10
SHMT1	1.300816509	2.86418E-07	10
FGGY	1.285840092	5.35326E-07	10
MSR1	1.242798432	2.08172E-06	9
C1R	1.221672189	1.14119E-05	8
PXDC1	1.215970564	4.78705E-06	10
SNX10	1.180155059	3.22888E-06	10
EGR2	1.159813019	2.75684E-05	9
ECM2	1.143890258	2.85371E-05	8
RARRES2	1.056811824	0.000141107	8
SIK1	1.050069253	4.08135E-05	9
ENPP2	-1.034842225	6.93985E-05	1
GALNT12	-1.081251536	2.79907E-05	1
CD248	-1.097230003	2.30987E-05	1
HSPA2	-1.1820773	1.02848E-05	1
TMEM119	-1.24777911	3.96659E-06	1
LAMA4	-1.368801109	7.79272E-08	0
SLC24A3	-1.419982393	1.29756E-07	0
GLT8D2	-1.541152586	6.34719E-09	1
CHN1	-1.638619619	6.9578E-10	0
OLFML2A	-1.706755646	1.62443E-10	1
MRGPRF	-2.080196958	2.24206E-13	0
HOXD8	-2.676747866	1.73791E-19	1
THBS4	-2.937992087	3.79912E-21	0
CRYAA	7.646594594	5.74829E-57	9
LPA	6.712901393	1.58914E-66	9
LOC100506229	6.505573463	1.85085E-50	9
SLC28A1	6.49612557	2.30094E-57	9
DBH	3.973605334	1.52124E-36	9
AR	2.90954714	4.86808E-20	9
HYAL1	1.854329272	4.11747E-10	8
ANGPTL4	1.772266101	1.29306E-10	9
PROX1	1.540499006	7.94331E-09	9
FBXO17	1.417910338	3.98813E-07	8
IL1RAP	1.369874118	1.17767E-07	9
HAAO	1.329282403	6.93413E-07	9
DCXR	1.20939621	2.23804E-06	9
FCN1	1.206620978	2.06023E-05	8
SHC2	1.203326232	5.29655E-06	8
TM4SF5	1.194370696	5.55106E-06	9
TFPI	1.174896156	1.16816E-05	8
SLC16A2	1.160406478	2.93149E-05	7
NADKD1	1.149516486	5.82066E-06	9
C1orf162	1.135957848	1.20262E-05	9
PRAP1	1.118060915	5.13591E-05	8

HMOX1	1.093557531	2.01979E-05	9
DNALI1	1.07412051	0.00010137	8
PEMT	1.03995902	3.39278E-05	9
FCGR2B	1.030280692	0.000130815	8
GLYCTK	1.024029959	7.90749E-05	8
PECR	1.022280636	6.32814E-05	8
GADD45B	1.01221475	8.91945E-05	8
PTGIS	1.006623405	0.000265	7
CDC42EP5	-1.006998455	0.000124525	1
RGS5	-1.008555452	7.72547E-05	1
MYL9	-1.012906933	0.000134294	2
TTC39A	-1.028554878	0.000112401	1
CH25H	-1.135551368	4.06631E-05	1
MXRA5	-1.153287872	1.37199E-05	0
SDR16C5	-1.17285989	0.000121185	2
HOXA6	-1.341430487	7.0874E-06	1
WFDC1	-1.342422848	2.47562E-07	0
DDR2	-1.364268014	3.55493E-07	1
C1QTNF3	-1.390483569	5.8868E-07	0
S100B	-1.440078042	3.49397E-07	0
CREB3L1	-1.612408635	4.78497E-10	0
SMAD9	-1.667633748	2.39306E-10	0
FGF7	-1.723608466	4.48481E-10	1
NDNF	-1.816214271	5.11852E-11	1
CAPN6	-1.838831317	3.43536E-09	2
MUC17	-1.93909586	6.21245E-09	2
RASD2	-2.348906274	7.01694E-17	1
MAB21L2	-3.194628406	1.99231E-24	0
HOXD13	-3.893212785	4.35266E-19	2
LINC00578	-5.018883752	2.06318E-42	0
FAM99B	9.275645489	2.10893E-63	8
AGXT2L1	7.807767695	1.94982E-62	8
APOC4-APOC2	7.670720567	2.25986E-26	8
SLC22A9	7.086937451	8.10374E-56	8
ANXA10	5.33346961	1.62279E-43	8
SLC1A2	4.766612932	2.11318E-45	8
CNDP1	3.944450458	1.46599E-28	8
THPO	3.788449448	6.90326E-27	8
KMO	2.790794121	2.02504E-21	8
ANGPTL6	2.747613659	1.3171E-20	8
FST	2.432610529	4.67375E-17	8
ETNK2	2.127196099	3.69034E-14	8
GRB14	1.992911528	9.65431E-12	8
SMOC1	1.965799562	3.81947E-11	8
CHST13	1.636757778	3.83765E-09	8
CGNL1	1.622005592	7.10003E-10	8
LOXL4	1.548693457	2.54773E-08	7
CHRD	1.526577282	1.64788E-08	8
DHODH	1.432963334	1.73049E-08	8
FAH	1.38225446	3.91194E-08	8
TMEM45A	1.355203203	5.84674E-07	8
ABLIM3	1.296956951	9.92122E-07	8
GPAM	1.218545782	2.0232E-06	8
CADM1	1.050357469	8.803E-05	7
STEAP3	1.036939033	3.23669E-05	8
PPP1R3C	1.031990592	0.000478846	6
ACOX2	1.026849769	4.85626E-05	8
PILRA	1.020078728	7.04686E-05	8
MACC1	0.242277919	0.482792188	5

ANGPTL2	-1.02879249	8.88501E-05	1
NEXN	-1.033837019	8.82822E-05	1
AFAP1L2	-1.080045837	1.63589E-05	0
KIAA1324	-1.094684453	7.19648E-05	1
HES2	-1.198422859	2.0786E-05	1
GAS7	-1.20396518	4.76674E-06	1
SSPN	-1.207014391	4.15601E-06	0
CPE	-1.220301264	2.21046E-06	0
SYNPO2	-1.281161567	4.24555E-06	1
COL9A2	-1.317527758	1.38372E-06	1
C11orf93	-1.330154695	3.03599E-06	1
WNT5B	-1.344313439	1.28594E-06	1
AKAP5	-1.37120561	6.70411E-07	1
OXCT1	-1.432315927	1.66162E-08	0
PDE5A	-1.437404727	2.35906E-08	0
GULP1	-1.64154172	6.03512E-10	0
PCDH7	-1.682017876	1.98536E-09	0
SERTAD4	-2.50568464	3.22792E-16	0
HOXD9	-2.633422733	6.89614E-17	0
TAC1	-2.950834946	4.10021E-18	0
CHRNA3	-3.656479926	1.28062E-26	0
PAQR9	8.355817439	7.48375E-54	7
CA5A	8.340452173	5.32521E-56	7
SLC17A3	6.652600386	1.59622E-46	7
OXT	2.955432691	1.08302E-13	7
GLT1D1	2.506920301	1.8238E-16	7
TMEM27	2.484707169	4.88403E-17	7
ADCY1	2.129654903	4.523E-14	7
TNFSF14	1.967039197	2.39505E-13	7
ENO3	1.44145112	2.97563E-08	7
NAGS	1.354482121	2.31751E-07	7
GPR37	1.300070415	7.906E-06	6
TTC39C	1.278167394	8.30682E-07	7
FBXO2	1.262826696	3.47767E-06	7
HS3ST3B1	1.224762872	1.05502E-05	7
TNFRSF11B	1.205786175	4.15662E-06	7
OSCAR	1.14896003	1.08204E-05	7
SLC23A2	1.111182836	1.64782E-05	7
HK3	1.061687293	2.92222E-05	7
SLC25A42	1.021092679	8.72536E-05	7
LGR6	1.014532799	0.000248374	5
CYGB	-1.000180701	9.02391E-05	0
TM4SF20	-1.006684666	0.000587268	1
NOV	-1.00859387	0.000178274	0
FILIP1L	-1.00998759	7.93628E-05	0
HIST1H2AI	-1.042490278	0.000537876	2
HOXA5	-1.045311418	0.000109109	0
SNCG	-1.088395389	0.000103848	0
PPP1R12B	-1.121530086	1.21557E-05	0
HOXA-AS3	-1.134941671	0.000112507	1
FGFR1	-1.149269071	1.07718E-05	1
PDE4D	-1.190141295	2.8126E-06	0
MEIS1	-1.270383681	2.62808E-06	0
CSPG4	-1.311719502	7.86568E-07	1
KCNMB1	-1.312921451	5.08613E-06	0
PPP1R36	-1.43719728	2.02485E-07	0
L1TD1	-1.49443532	8.76003E-07	1
HOTTIP	-1.59228212	2.21801E-07	1
HOXA11	-1.679031991	1.32432E-09	0

CA8	-1.684763984	3.31162E-08	0
OSR2	-1.973727791	2.13423E-12	0
LEFTY2	-2.173378838	6.85848E-12	0
C1orf133	-2.284200531	6.71643E-13	0
BOC	-2.315656733	2.07473E-15	0
DIO2	-2.552189505	5.10659E-17	0
BBOX1	5.067015285	1.77289E-34	6
MLIP	3.591634775	2.19628E-22	6
PFKFB1	3.287391424	3.13916E-25	6
SLC7A9	3.020883705	1.03217E-18	6
PANX2	2.283004062	9.78296E-13	6
HS3ST2	2.145281875	7.74478E-13	6
ADRA1B	2.113125632	2.6131E-13	6
CCL17	1.949512314	3.45242E-07	6
BCO2	1.94680659	9.96338E-12	6
LIN7A	1.780385739	9.60907E-11	6
AVPR1A	1.666293906	1.26171E-07	6
DTX1	1.40086917	1.19838E-07	6
RETN	1.37977879	6.16334E-05	6
FOLR1	1.303790306	6.02595E-05	5
ST3GAL6	1.241344802	3.87317E-06	6
ABCC6	1.182723868	7.15858E-06	5
RTN4RL2	1.102403645	9.03529E-05	5
OSGIN1	1.026322436	6.74486E-05	6
C13orf33	-1.002783483	0.000238233	1
FOXL1	-1.04939636	9.11615E-05	1
TCEAL7	-1.062428385	0.000155355	1
ZC3H12C	-1.137940987	1.80639E-05	1
TPBG	-1.150895155	7.0099E-06	0
SPECC1	-1.162926607	4.41403E-06	0
BDKRB2	-1.163824165	3.68204E-06	0
BMP8A	-1.192656674	5.31988E-06	0
A4GALT	-1.223097428	3.96776E-06	0
SYNM	-1.380648189	5.29667E-07	0
PRUNE2	-1.478226671	5.11874E-08	0
EPHA4	-1.636058055	5.37402E-09	0
FLNC	-1.642030874	8.15273E-10	0
LOC375295	-1.67409606	1.0658E-07	1
ADAMTS14	-1.680696262	1.664E-10	0
SPEG	-1.803438456	6.21175E-10	0
COL4A5	-1.817096006	2.25475E-10	1
C11orf92	-1.855509809	1.62242E-10	1
HOXD-AS2	-1.865259492	6.19022E-08	1
FOXP2	-1.922329109	4.15869E-11	0
JPH2	-2.024904014	2.7889E-12	0
COL9A1	-2.446410986	1.67687E-16	0
PCSK1	-2.80368147	4.51538E-19	0
LOC728040	8.36127929	7.1917E-47	5
CHRNA4	6.657774484	7.27669E-52	5
TRPM8	6.509627887	3.04441E-60	5
ONECUT1	6.000191625	1.47777E-46	5
FOLH1B	5.793559865	1.17676E-40	5
GPR182	3.45540999	4.73941E-23	5
SULT1E1	2.80793185	8.53154E-17	5
CYP2C19	2.597728677	7.34472E-15	5
PNMA6C	2.355502971	2.81005E-11	5
GPLD1	2.120498225	1.40327E-13	5
SLC1A3	1.562187215	1.39322E-08	5
DNAJC5B	1.523359502	2.62318E-07	5

BMPER	1.307873251	1.09254E-05	5
HSPA6	1.274921868	2.10225E-06	5
P2RX7	1.218680672	4.37818E-06	5
SIGLEC9	1.163848993	7.02413E-06	5
KDM8	1.085907363	2.16466E-05	5
SLC2A9	1.063727855	2.26401E-05	5
PIEZO2	1.024192475	0.000146071	4
PTPRN2	-1.059771416	4.82534E-05	0
PLA2G4A	-1.102829023	2.03799E-05	1
ARSJ	-1.1652657	1.12991E-05	1
GPR68	-1.207616813	4.15555E-06	1
RASL12	-1.254021333	1.87749E-06	0
C3orf70	-1.326094922	4.40603E-07	0
HSPB7	-1.478099741	1.12269E-06	0
CCDC48	-1.56351223	3.28547E-09	0
SGCD	-1.573356056	2.80073E-09	0
HOXA11-AS	-1.59772336	1.03148E-08	0
MIR143HG	-1.669098358	8.17744E-10	0
SCRG1	-1.72152003	2.2346E-05	0
LSAMP	-2.468338008	1.4772E-17	0
SYNDIG1	-3.198527908	2.13392E-24	0
LRRN4CL	-3.211903111	1.23958E-26	0
FGF10	-3.840127452	1.30414E-19	0
LOC100507389	8.069371555	3.46405E-49	4
SLC6A13	5.725855129	3.8608E-35	4
EPO	5.574486683	6.03004E-35	4
ENPP7	5.507295399	1.77791E-36	4
C22orf45	3.547492443	8.76975E-27	4
RANBP3L	3.308899021	6.25225E-18	4
RIPPLY1	3.178067815	3.60832E-15	4
AOC4	2.877501777	3.24306E-14	4
CES1P1	2.778096104	3.67613E-13	4
HTR2B	2.124798814	3.26973E-11	3
PRAME	2.036993446	7.69239E-07	4
CHIT1	1.903677971	5.07432E-09	3
CST6	1.636164808	1.11538E-05	4
KCNN2	1.563246541	1.01915E-07	4
CXCR2P1	1.478004704	4.39408E-07	4
CACNG4	1.4353087	1.96921E-05	4
FITM1	1.393029047	2.56164E-06	4
SLC16A11	1.352531244	7.69037E-07	4
SUCNR1	1.310357099	3.21512E-06	4
AQP7P3	1.226469274	0.000122868	4
ADAMTS13	1.162119229	5.04515E-06	4
HAS2-AS1	1.058427703	0.000562511	4
LILRA2	1.057833514	0.000110107	4
CYP4F11	1.04635221	5.101E-05	4
AKR7L	1.046275661	0.000114266	4
DFNA5	1.008896814	0.000109153	4
DLL1	-1.009114479	5.25422E-05	0
SELP	-1.038448587	0.000143601	0
POPDC2	-1.077066227	0.000147395	0
EFS	-1.12032958	2.8602E-05	0
SNPH	-1.21250496	9.17898E-06	1
SCARA3	-1.223575831	3.8293E-06	0
GATA2	-1.294142468	4.35106E-07	0
HOXA13	-1.383162696	1.12472E-06	0
PSD	-1.49867088	4.06125E-07	0
MEOX1	-1.568329553	1.15932E-08	0

TACR2	-1.5848975	1.48189E-06	0
VSTM5	-1.801845795	9.02874E-08	0
CSDC2	-2.019184485	2.33883E-10	0
ADCYAP1	-3.253833739	1.86633E-21	0
CYP1A1	7.282442885	4.65831E-44	3
SLC34A2	5.766673933	2.03822E-44	3
CYP26A1	3.411226092	1.57444E-18	3
LRAT	3.242748688	8.92739E-20	3
CLDN10	3.153590254	4.18006E-19	3
LOC728218	2.914772387	2.2159E-12	3
ZNF385B	2.094638739	5.3452E-10	3
TNFSF18	1.78670192	1.8752E-07	3
VWCE	1.745905146	7.19709E-09	3
GPR88	1.698522572	1.26403E-06	3
LPPR1	1.660679116	3.40746E-07	2
KCNJ5	1.607939613	2.05141E-08	3
HAS1	1.591965025	3.28815E-06	2
IP6K3	1.552469896	5.89274E-07	3
PPP1R1C	1.494788557	1.5555E-06	3
ZBED2	1.348389934	3.01043E-05	2
ALPK2	1.309556331	1.9069E-06	3
PHYHIPL	1.243615212	3.51505E-05	2
NTS	1.235330588	0.000332412	2
MUC6	1.223895594	0.000177611	2
TNNT1	1.160424415	0.000645694	2
EFHA2	1.153228762	4.76252E-05	3
PLA2G5	1.030719936	0.000685779	1
EOMES	1.018846963	0.000423247	3
MFAP3L	1.017221566	7.14163E-05	3
CRMP1	-1.001294276	0.000101104	0
AIF1L	-1.01002431	0.000123336	0
HOXA7	-1.036886291	0.000200162	0
CTF1	-1.057367391	0.000263723	0
CNTNAP1	-1.069985033	3.15378E-05	0
ARSI	-1.22275846	1.25851E-05	0
ST6GALNAC5	-1.238183373	1.88939E-05	1
ROR1	-1.246814859	2.05683E-05	0
PDGFRL	-1.264125085	9.70142E-06	1
FAM155A	-1.278516593	2.05657E-05	1
SLC16A14	-1.283583929	8.32337E-07	0
GLI3	-1.30495373	2.74175E-06	0
LOC100127983	-1.346253376	0.000170063	0
CYTL1	-1.386611347	6.61346E-06	0
TNIP3	-1.544608589	2.80625E-07	0
ZNF521	-1.553581173	8.49521E-09	0
TMTC1	-1.654342003	1.11901E-09	0
MEOX2	-1.724779218	2.50667E-08	0
CYP26B1	-1.785179137	2.09637E-10	0
LOC389493	-1.814540927	3.94833E-05	1
PTGER3	-1.924779226	7.10023E-11	0
SDK1	-2.527501233	2.00107E-18	0
MYOCD	-2.694543048	6.54526E-20	0
GDNF	-2.812355002	2.51106E-16	1
TCEAL2	-3.098437411	1.90415E-13	0
OR51E2	-3.22898753	5.02536E-20	0
NKX3-2	-3.259399864	1.13649E-19	0
HOXD12	-3.455676632	2.01505E-12	0
CARTPT	-5.391533427	2.29417E-15	0
SLC22A25	9.086716305	9.50419E-41	2

BMP10	6.852087564	1.56539E-32	2
KRT16P3	6.710724152	1.47093E-14	2
COX6A2	5.322401972	3.20944E-10	2
AKR1CL1	5.306457243	3.00757E-27	2
RHBG	5.065510329	3.11441E-28	2
LOC100507055	4.41372797	1.21914E-31	2
LOC100131726	4.128063524	6.42277E-17	2
LINC00313	3.384139727	3.01629E-18	2
LHX2	3.132483752	1.47236E-20	2
ITGAD	3.021433777	2.27734E-22	2
CYP4F22	2.946220784	1.80451E-19	2
ARID3C	2.837676247	3.75605E-16	2
TPPP2	2.827122861	4.19044E-13	2
IDO2	2.606472478	4.76917E-09	2
IL27	2.5776969	1.16779E-12	2
LOC145820	2.501062845	1.99171E-14	2
LOC645434	2.295509923	2.78482E-05	2
FAM35B2	2.173547688	1.83611E-09	2
TCP10L	1.993508849	9.76707E-10	2
PMCH	1.759325134	0.000279615	2
GLUD2	1.696645402	1.69255E-08	2
RSPO4	1.6531766	0.000410446	2
DKFZp779M0652	1.385343145	0.000212848	2
RDH12	1.272288826	4.44293E-05	2
SIGLEC11	1.157371706	6.16393E-05	2
CD226	1.029685217	9.58655E-05	2
RAB9B	-1.035803028	0.000280668	0
KIAA1644	-1.049495353	0.000227398	0
SBSPON	-1.071311374	0.000140806	0
FAM167A	-1.148550108	2.27926E-05	0
PART1	-1.154180483	0.000377397	0
ANKRD65	-1.184246499	3.6774E-05	0
PRDM6	-1.197379204	6.22688E-05	0
COX4I2	-1.224903966	0.000146042	0
FGF2	-1.250959468	7.26758E-06	0
GNAL	-1.254958739	2.13008E-06	0
SLC8A2	-1.264185233	0.000499787	0
ENOX1	-1.331891795	3.62451E-06	0
COCH	-1.347380889	5.14625E-06	0
RET	-1.371113817	1.64933E-06	0
KL	-1.382800491	2.84884E-06	1
IL26	-1.43372351	0.000924375	0
PRRX2	-1.453492687	1.48339E-06	0
PTPRN	-1.45947183	1.15174E-06	0
CEBPE	-1.513174315	2.15415E-06	0
KBTBD12	-1.519727658	1.29019E-07	0
DOC2B	-1.54054218	1.23997E-07	0
FBXL22	-1.541226722	6.15196E-08	0
LPAR3	-1.556659607	2.14499E-05	1
LRCH2	-1.613624403	5.55969E-08	0
CHI3L2	-1.61494107	3.69067E-07	0
CLDN18	-1.653530787	0.00024943	0
BHLHE22	-1.662223929	3.35144E-09	0
COL28A1	-1.7240932	4.97327E-10	0
BNC2	-1.802002968	1.27585E-10	0
WNT9A	-1.820920348	4.20355E-09	0
IGDCC4	-1.824634757	2.79031E-11	0
TAC3	-1.837997633	3.36991E-08	0
HDC	-1.880781677	2.14352E-10	0

ADAMTS16	-1.886047332	2.30873E-09	0
PTGFR	-2.016890682	1.30015E-12	0
FHL5	-2.355266762	1.80618E-14	0
VIPR2	-2.470968474	8.71269E-16	0
ADAMTS8	-2.815810734	2.66742E-20	0
FBN2	-2.965015344	1.0438E-25	0
ASB5	-7.122683553	8.2819E-17	0
FLJ42280	8.946809188	4.63538E-17	1
PRAMEF10	8.79327267	3.56154E-14	1
SERPINA12	7.472124595	2.50325E-25	1
UGT1A5	6.561388243	1.46167E-06	1
LOC157273	6.515687608	1.70976E-25	1
BASP1P1	5.840275403	4.23198E-05	1
MT1B	5.754308844	9.69601E-05	1
GOLGA6B	5.023509486	2.75815E-15	1
CES5A	4.94031015	1.57006E-17	1
CYP3A7-CYP3AP1	4.921319826	6.05772E-08	1
TAKR	4.821147014	6.99106E-24	1
LOC643037	4.649821128	8.15223E-06	1
ARSF	4.523883364	1.62102E-14	1
PRR18	4.507033386	6.95221E-25	1
GOLGA6C	4.490597573	6.24148E-23	1
KRT16P2	4.425705558	1.03456E-10	1
LHX9	4.1510903	3.52393E-13	1
PIK3C2G	3.986777508	6.15908E-30	1
GMNC	3.834102986	1.07091E-14	1
PTH2R	3.636941008	4.33707E-13	0
UPP2	3.589468063	3.79017E-11	1
GOLGA6A	3.527885236	3.56515E-09	1
LOC100128675	3.299810324	1.94436E-11	1
ANKRD1	3.255198671	1.2969E-18	1
PKHD1	3.170799572	5.1938E-25	1
DGCR5	2.963261216	5.18345E-18	1
ZIC1	2.951118112	4.30815E-11	1
SYT9	2.892008526	1.97517E-13	1
ACOT6	2.765854115	2.3628E-09	1
DNMT3L	2.675287799	1.2671E-09	0
EMID2	2.621623934	1.1457E-13	1
LOC401109	2.43520783	8.085E-06	1
IGF2BP1	2.193877911	2.61041E-09	1
LOC200772	2.058563118	1.76297E-09	1
GREB1L	2.04144946	2.91421E-10	1
PROX1-AS1	2.038766743	5.71283E-12	1
C8orf46	2.02635547	1.17305E-07	1
MCHR1	2.006300037	2.82481E-10	1
NALCN	1.982974037	1.31294E-11	1
LOC284260	1.919696423	0.000348177	1
WNK3	1.808977489	1.38861E-08	1
MRO	1.79466379	6.9444E-10	1
PRSS35	1.773517053	6.47577E-10	1
LOC100133669	1.740117961	8.61778E-07	1
HAPLN4	1.739050839	4.24574E-08	1
ABCC11	1.728075907	1.53275E-07	1
PPP4R4	1.723457097	3.21421E-07	1
CECR2	1.683295187	2.42908E-09	1
CYP11A1	1.460321171	1.01105E-05	1
CHRNE	1.405357963	3.74909E-06	1
SLC6A16	1.370894571	5.53441E-06	1
LINC00478	1.354835338	2.43318E-05	1

KCNJ10	1.34956922	5.0141E-06	1
NPR3	1.290315952	7.41975E-06	1
B3GAT1	1.260944713	2.18723E-05	1
RNF165	1.259562874	8.33591E-05	1
HSPB9	1.245432908	0.000281367	1
CD1B	1.21045483	0.00056507	1
LOC100130015	1.199270759	2.54702E-05	1
ZNF365	1.166704237	0.000318342	1
TNNT2	1.150028016	0.000128599	1
NXF3	1.117865581	0.000743204	0
CA14	1.112722522	0.000185532	1
SOX5	1.087634755	7.82848E-05	1
TMPRSS9	1.079416138	0.000154579	1
PRAM1	1.046316424	6.47909E-05	1
PNMAL1	-1.003942559	0.000456367	0
KCNN3	-1.020133277	0.000151827	0
ARHGEF4	-1.0234014	0.000214164	0
FAM163A	-1.026119638	0.000435048	0
EBF1	-1.026972981	0.000106648	0
TRIM9	-1.029967552	0.000482867	0
TRAM1L1	-1.032126842	0.00063776	0
PTGER1	-1.035436409	0.000650049	0
BEND6	-1.042828301	0.000919357	0
NCAM2	-1.060390542	0.000294373	0
OR51E1	-1.067784227	0.000170938	0
SDK2	-1.072471423	0.000104491	0
RUNX1T1	-1.088593425	5.16822E-05	0
C2CD4C	-1.100473849	0.000293637	0
REM1	-1.109356351	0.000171515	0
NACAD	-1.130440944	6.99378E-05	0
CEND1	-1.135544479	0.000857715	0
ELOVL4	-1.187305733	4.6679E-05	0
STK32B	-1.187928471	2.26239E-05	0
HRASLS5	-1.240650215	0.000929715	0
PCDHB2	-1.263695279	6.07427E-06	0
PREX2	-1.289999185	2.62522E-06	0
SLC25A48	-1.311903289	7.53904E-05	0
FAT4	-1.361531279	3.6072E-07	0
TRPC6	-1.381640433	5.36259E-07	0
APLP1	-1.382056117	1.21904E-06	0
FFAR2	-1.396430095	2.54912E-07	1
RAB3C	-1.40398901	6.33772E-05	0
NUDT11	-1.444230964	2.18669E-06	0
PLXNA4	-1.455768365	1.9284E-07	0
PPAPDC3	-1.465945884	6.23613E-07	0
LOC650368	-1.47063549	2.86303E-06	0
CHRNB4	-1.538631363	5.39229E-05	0
C3orf36	-1.547561086	8.84931E-07	0
ADAMTS19	-1.550510385	6.86922E-05	1
CCDC85A	-1.5737176	5.70433E-08	0
KCNV1	-1.605565027	1.40633E-05	0
AK5	-1.606452449	1.28631E-06	0
ARHGAP28	-1.634712405	1.40538E-08	0
EGF	-1.650727916	1.78175E-07	0
SORCS2	-1.652625652	1.70926E-09	0
C1QTNF9	-1.675345765	4.95719E-06	0
KIRREL3	-1.694611467	3.50077E-07	0
ADAMTS18	-1.718008908	9.52279E-06	0
IL17B	-1.745643656	2.87251E-05	0

AMPH	-1.7703314	1.00202E-09	0
DPY19L2	-1.77840608	2.4983E-08	0
LPPR4	-1.79366699	2.38569E-10	0
LOC146336	-1.809045002	1.89875E-06	0
C15orf59	-1.829172937	2.58644E-09	0
PTPN5	-1.865176228	2.98998E-08	0
EFHC2	-1.871011599	3.60914E-09	0
AVPR2	-1.876021447	4.38779E-09	0
COL24A1	-1.890014947	1.26363E-10	0
ZNF385D	-1.915677835	8.92875E-09	0
ODZ3	-1.916048779	9.99765E-10	0
SLC35F1	-2.050103058	3.05375E-10	0
PDZD2	-2.129909496	4.54486E-15	0
NTNG1	-2.181116235	1.80936E-05	0
LGALS12	-2.296930104	1.00876E-10	0
DGKB	-2.329500678	8.72783E-09	0
TACR1	-2.351101978	1.01125E-09	0
TLL1	-2.407422127	1.24106E-14	0
MKX	-2.442834339	1.332E-16	0
DRD5	-2.488458066	0.000173164	0
SLC18A3	-2.55237522	0.00052151	0
EPHA6	-2.574697505	3.60382E-06	0
GRIA1	-2.65056194	1.0636E-08	0
SOX2	-2.756585947	1.08604E-10	0
NOS1	-2.884119825	5.97827E-12	0
EBF3	-2.949477083	1.50196E-19	0
AARD	-3.016916904	2.27365E-11	0
PDE1C	-3.075222538	8.41481E-22	0
LOC100131825	-3.148264774	2.78386E-11	0
LRFN5	-3.227416962	1.85525E-15	0
LMO1	-3.394058962	4.52809E-05	0
SLITRK5	-3.745082279	1.59645E-16	0
GALNTL6	-3.831469909	1.31826E-17	0
MAGEB2	-4.194574331	0.000163352	0
IL22	-4.503041278	1.54016E-07	0
PENK	-5.133206981	1.49831E-12	0
GSTA7P	9.213895131	3.05912E-18	0
FAM9B	9.122804377	2.42051E-18	0
LIN28B	7.944499212	5.5566E-06	0
HOXC13	7.264125575	2.62261E-06	0
LOC100506409	7.178236155	2.02733E-09	0
LOC286370	6.864973226	5.86612E-07	0
MIR4500HG	6.68231876	8.59211E-07	0
ZNF716	6.122789122	0.00050647	0
C6orf7	6.113123439	2.87524E-05	0
PSG5	5.997119873	0.000106587	0
LOC339685	5.915052493	3.18451E-05	0
HORMAD2	5.828776697	3.66663E-10	0
FGF14-IT1	5.705293022	0.000136532	0
HOXC12	5.636667778	0.000454889	0
CYP2A13	5.419155812	0.000439928	0
MS4A6E	5.215142921	0.000662342	0
APOL5	4.790217786	4.76004E-14	0
DLK1	4.722515838	1.861E-09	0
TRPC5	4.499890303	3.81774E-12	0
FBXL21	4.265265288	1.57816E-05	0
PNPLA5	4.209046562	2.41913E-05	0
GNAT1	4.203868904	3.54127E-08	0
LOC255480	4.004502043	0.000113615	0

NAV2-AS4	3.938137812	0.000473475	0
ADH7	3.911366404	0.000696204	0
DEFB132	3.880412533	6.95291E-05	0
PCDP1	3.818581402	1.59314E-20	0
LYPD2	3.737659328	5.62709E-12	0
ALLC	3.718631562	4.06278E-11	0
LOC154092	3.515903033	1.13072E-17	0
SPIC	3.482501	5.34854E-10	0
RGSL1	3.403928554	3.60867E-07	0
NPPB	3.341611105	1.27773E-05	0
SEC14L3	3.336090457	2.47387E-07	0
LOC388387	3.215478913	2.37742E-13	0
IL36B	3.209377814	2.35363E-06	0
SPATA21	3.20759155	2.08426E-11	0
HEATR7B2	3.179054432	3.23678E-06	0
ZIC4	3.173469973	5.57612E-05	0
WDR65	3.13253104	6.74647E-21	0
FRMD7	3.049586708	0.000903559	0
PRTN3	3.0134669	0.000178076	0
ALDH1L1-AS1	3.005758942	1.98132E-05	0
MYH6	3.005179093	2.58603E-05	0
CYP17A1	3.001524284	2.57893E-14	0
ZCCHC16	2.909478283	4.18804E-06	0
DCDC5	2.8434687	9.79646E-10	0
LOC100131208	2.742205947	4.69029E-07	0
DPPA4	2.689677642	5.58149E-05	0
SLC6A1-AS1	2.686360279	2.38203E-06	0
RBM46	2.680297212	0.000351524	0
ZAN	2.662491229	0.000490712	0
C21orf37	2.653446388	2.41698E-07	0
RTL1	2.644060239	1.38876E-05	0
EPHA8	2.637414173	1.62911E-05	0
LOC100133308	2.600556797	1.56469E-05	0
LINC00563	2.600233966	0.000239748	0
RNASE13	2.595884735	3.74005E-06	0
C9orf106	2.568353046	9.35522E-05	0
C1orf98	2.545514501	1.28052E-05	0
LOC100506085	2.534128048	1.96623E-08	0
IGF2-AS	2.509079419	2.21308E-07	0
ADCY10	2.457414586	5.04798E-13	0
AQP4	2.440185726	5.47921E-06	0
THSD7B	2.409530661	9.24963E-12	0
A4GNT	2.403766132	0.000510155	0
LOC339240	2.386748691	1.28201E-06	0
MGC27382	2.360279517	2.2024E-06	0
AOX2P	2.348282768	0.000238507	0
KRTAP5-6	2.344007531	0.000275801	0
USH2A	2.336258776	5.81755E-11	0
FAM35B	2.198474456	3.63517E-07	0
EMBP1	2.188371842	2.47692E-09	0
C2orf91	2.126425288	4.55586E-06	0
OR5AK4P	2.10874459	0.000582951	0
ZPLD1	2.106640976	0.000575665	0
ALOX12P2	2.027570493	2.51776E-08	0
LINC00470	2.008819902	0.000224285	0
NRG3	2.008125799	6.84738E-09	0
CYP4F8	1.998785027	1.01879E-05	0
TMEM132E	1.972415762	1.46737E-11	0
CYP4Z1	1.957327365	0.000344439	0

RXFP1	1.921792471	1.52496E-11	0
ANO3	1.910807889	1.21238E-07	0
COL25A1	1.89423469	8.8506E-08	0
SLC34A1	1.887891358	0.00037686	0
LOC284551	1.874581984	0.00069186	0
WNT7A	1.86401342	0.000600492	0
AMZ1	1.846691763	9.17909E-09	0
LOC100130539	1.806817975	0.000136463	0
KNDC1	1.751347609	2.4173E-10	0
ESPNP	1.747859881	2.47764E-08	0
MYBPH	1.734821016	0.00015618	0
FBN3	1.689696681	0.000125435	0
PTCRA	1.683981533	1.16493E-05	0
TNN	1.67977646	3.82007E-09	0
ENTHD1	1.656731869	4.56687E-05	0
RGPD1	1.613788644	0.00027315	0
ANKRD55	1.581910227	9.53822E-07	0
SOAT2	1.542047444	0.000101334	0
CLEC4C	1.538674233	0.000872987	0
LGSN	1.496556504	3.28737E-05	0
POU3F1	1.493115433	0.000456878	0
C12orf59	1.483899437	7.64637E-06	0
GRM6	1.460126512	0.000334924	0
GATA5	1.415567855	0.00014305	0
KCNE1	1.414592406	0.000122176	0
MYO16	1.407799833	1.9323E-05	0
ANO4	1.400357983	7.29454E-06	0
GOLGA8IP	1.380001018	9.15279E-05	0
FCRLB	1.37731945	1.85401E-06	0
IFITM10	1.359450464	1.42074E-07	0
KEL	1.300807754	9.07717E-05	0
LINC00238	1.299333002	0.00016319	0
C9orf139	1.285603383	3.25176E-05	0
KRT5	1.282453034	0.00038673	0
TCHH	1.281523409	1.22063E-05	0
TPRG1	1.271270609	1.85308E-05	0
PDE6G	1.23998328	1.86325E-05	0
FOXC2	1.214134631	1.67566E-05	0
LRRC4C	1.202436782	3.60092E-05	0
C5orf49	1.197811907	0.000625555	0
GPR77	1.18754564	2.24795E-05	0
FAM43B	1.174397433	0.000444358	0
POM121L9P	1.07779538	0.000130569	0
LOC100507472	1.030088007	0.000481383	0
PKD2L1	1.015962809	0.000967274	0
CCDC89	-1.005338272	0.00093721	0
CACNA2D3	-1.018637789	0.000730888	0
KIAA1377	-1.043636741	5.14204E-05	0
MYCBPAP	-1.072396075	0.00068256	0
JPH3	-1.081115772	0.000523107	0
PRPH2	-1.086896204	0.000330359	0
NWD1	-1.087532128	0.000366666	0
OPCML	-1.110341211	0.000871865	0
RGS17	-1.110772096	0.000965671	0
GPR20	-1.124564984	0.000230091	0
GRIP2	-1.158121796	1.84052E-05	0
KSR2	-1.158450467	9.03981E-05	0
ROS1	-1.15937339	0.000660063	0
LOC100292680	-1.218317762	0.000369877	0

LHFPL3	-1.237093249	0.000126747	0
CHST6	-1.241644747	6.62159E-06	0
NRK	-1.254022547	0.000182513	0
FSTL4	-1.270994598	0.000609817	0
EFCAB5	-1.276300007	1.49912E-05	0
NPHS1	-1.293367564	0.000485366	0
OTOGL	-1.334909488	0.00029852	0
CATSPERB	-1.337709005	1.97035E-05	0
SLC10A4	-1.337900622	0.000585196	0
PAK3	-1.366273336	2.55476E-05	0
KIRREL2	-1.368819638	0.000726259	0
ZSCAN23	-1.370005402	5.00969E-05	0
ST8SIA1	-1.381290462	2.41909E-06	0
CCDC60	-1.406573001	0.000188871	0
RIMBP2	-1.413071562	1.18899E-06	0
PPFIA2	-1.432106502	1.76184E-06	0
RAB44	-1.486355708	8.47876E-07	0
AJAP1	-1.52100193	4.61799E-06	0
AP3B2	-1.600715461	9.146E-07	0
LRRTM2	-1.608788391	1.60463E-06	0
SCN3B	-1.629004952	2.3012E-07	0
FOXN1	-1.63893264	1.26841E-05	0
NPPC	-1.644824641	0.000235391	0
TMEM132B	-1.663895643	1.61688E-07	0
DAND5	-1.691789602	0.000123525	0
GRIK4	-1.702401389	7.84821E-06	0
STAC2	-1.720194867	2.36377E-05	0
FAM216B	-1.746864976	0.000865359	0
LOC100507632	-1.749859423	9.71919E-05	0
GPR63	-1.758163788	2.34182E-10	0
GP9	-1.805146819	0.000341514	0
TLL2	-1.808533766	2.50415E-09	0
IL1RAPL1	-1.815143106	0.000656088	0
NAP1L6	-1.832387481	0.000458509	0
LOC100507244	-1.837007724	0.000886964	0
COL6A5	-1.861145531	8.01587E-07	0
ANO2	-1.899607474	4.63205E-09	0
LOC401463	-1.909488315	7.71531E-05	0
RHBDL3	-1.916674486	2.58539E-09	0
ARMC4	-1.927628602	3.46679E-07	0
CACNA1G	-1.929107389	1.68774E-07	0
CYP27C1	-1.929357262	6.76252E-09	0
ST8SIA2	-1.939502572	1.44681E-06	0
STXBP5L	-1.948863453	1.63628E-06	0
RD3	-2.043408273	5.61198E-05	0
LOC285548	-2.048691225	9.19933E-07	0
MAFA	-2.070828221	9.54242E-05	0
ISL2	-2.072040592	3.55968E-10	0
GPR1	-2.093320559	2.07477E-08	0
LINC00461	-2.120212043	9.0265E-05	0
HOXC6	-2.133836098	3.44904E-05	0
KCNC1	-2.149233118	1.62132E-07	0
RPE65	-2.162699193	0.00021383	0
CDK15	-2.172305732	1.96232E-09	0
HTR2A	-2.182191884	4.91985E-10	0
FOXI2	-2.191395787	3.62831E-06	0
WFIKKN2	-2.211813558	3.1417E-07	0
PRRG3	-2.232269659	7.81778E-07	0
EYA4	-2.301751	6.80707E-10	0

LOC729950	-2.305537964	2.1751E-11	0
GLIS1	-2.313381645	1.78056E-09	0
DAZL	-2.329731163	3.38147E-05	0
NPFFR2	-2.334820767	8.5771E-06	0
LCN6	-2.339712232	1.52973E-08	0
LINGO2	-2.34903701	0.000262725	0
ATP2B3	-2.38973706	5.99073E-06	0
RTBDN	-2.391388168	1.18488E-05	0
KIAA0087	-2.420582523	8.15392E-06	0
NPAS4	-2.529796017	3.76308E-06	0
C20orf166	-2.585705715	0.000414921	0
C18orf34	-2.597629782	2.86524E-06	0
CLVS2	-2.638006566	5.46109E-05	0
TMEM229A	-2.640825773	4.35607E-06	0
LIX1	-2.652224179	3.38004E-06	0
FAM48B1	-2.699402156	0.000451942	0
HIST1H2BI	-2.789433537	2.24377E-05	0
SRRM4	-2.812412846	5.03249E-05	0
EMX2OS	-2.895351072	5.57155E-06	0
FRMPD4	-2.959911148	6.76146E-08	0
MYH13	-2.997624589	2.92517E-09	0
KCNB2	-3.069222451	2.13024E-08	0
NELL1	-3.085112951	5.13941E-13	0
IL19	-3.095520832	7.87557E-06	0
GRIA2	-3.161329887	4.39082E-06	0
CLCA3P	-3.183540818	1.98058E-05	0
FAM19A1	-3.276958533	3.64538E-14	0
MEIS1-AS3	-3.330403886	1.86817E-11	0
LOC644838	-3.372582786	1.40641E-05	0
TMEM215	-3.463202545	3.84891E-07	0
SNTG2	-3.51668836	3.87248E-10	0
LOC100129027	-3.61338668	0.000832299	0
ADCY2	-3.641761347	2.38462E-26	0
LOC286184	-3.657302474	0.000880406	0
C2orf71	-3.73111831	0.000734767	0
SYT14	-3.871791926	4.32639E-07	0
IFLTD1	-3.932559257	7.86609E-05	0
DCAF12L2	-3.954229275	7.83626E-09	0
GPR148	-3.962984218	0.000345843	0
CCKAR	-4.201167828	2.88702E-05	0
RIMS1	-4.290761684	3.65641E-16	0
KCTD8	-4.468417736	1.70678E-09	0
LOC150622	-4.473283039	3.30998E-05	0
FUT9	-4.511754473	3.94018E-05	0
KCNA1	-4.710076447	7.25195E-09	0
NXPH2	-4.836042665	1.78309E-07	0
PCDH15	-4.865046115	8.76745E-08	0
LPPR5	-5.109004947	2.23885E-09	0
SEL1L2	-5.347350428	0.000577961	0
FGF4	-5.560049279	0.00039362	0
PCDHB1	-5.696092102	0.000331071	0
EVX2	-5.771745143	1.25832E-11	0
OPRM1	-6.018823318	4.65827E-05	0
DBX2	-6.046994699	0.00022297	0
RP1-177G6.2	-6.347446014	1.70288E-05	0

Patient count_2-fold.downregı	Patient count_2-fold.ALL	Patient_ratio(%)
1	16	88.889
0	16	88.889
0	16	88.889
1	16	88.889
1	16	88.889
1	16	88.889
13	16	88.889
15	16	88.889
16	16	88.889
16	16	88.889
14	16	88.889
0	15	83.333
0	15	83.333
0	15	83.333
0	15	83.333
0	15	83.333
0	15	83.333
0	15	83.333
0	15	83.333
0	15	83.333
0	15	83.333
0	15	83.333
0	15	83.333
0	15	83.333
0	15	83.333
0	15	83.333
0	15	83.333
0	15	83.333
0	15	83.333
0	15	83.333
0	15	83.333
0	15	83.333
2	15	83.333
1	15	83.333
0	15	83.333
11	15	83.333
14	15	83.333
14	15	83.333
15	15	83.333
15	15	83.333
0	14	77.778
0	14	77.778
0	14	77.778
0	14	77.778
0	14	77.778
0	14	77.778
0	14	77.778
0	14	77.778
0	14	77.778
0	14	77.778
0	14	77.778
0	14	77.778
0	14	77.778
0	14	77.778
0	14	77.778
0	14	77.778
1	14	77.778
0	14	77.778

1	14	77.778
0	14	77.778
3	14	77.778
1	14	77.778
0	14	77.778
1	14	77.778
3	14	77.778
2	14	77.778
3	14	77.778
9	14	77.778
14	14	77.778
12	14	77.778
13	14	77.778
14	14	77.778
12	14	77.778
0	13	72.222
0	13	72.222
0	13	72.222
0	13	72.222
0	13	72.222
0	13	72.222
0	13	72.222
0	13	72.222
0	13	72.222
1	13	72.222
0	13	72.222
0	13	72.222
0	13	72.222
2	13	72.222
0	13	72.222
1	13	72.222
1	13	72.222
11	13	72.222
11	13	72.222
11	13	72.222
12	13	72.222
12	13	72.222
13	13	72.222
12	13	72.222
12	13	72.222
0	12	66.667
0	12	66.667
0	12	66.667
0	12	66.667
0	12	66.667
0	12	66.667
0	12	66.667
0	12	66.667
0	12	66.667
0	12	66.667
0	12	66.667
1	12	66.667
1	12	66.667
2	12	66.667
1	12	66.667
1	12	66.667
2	12	66.667
3	12	66.667
11	12	66.667

11	12	66.667
10	12	66.667
11	12	66.667
11	12	66.667
11	12	66.667
11	12	66.667
10	12	66.667
12	12	66.667
12	12	66.667
12	12	66.667
0	11	61.111
0	11	61.111
0	11	61.111
0	11	61.111
0	11	61.111
1	11	61.111
1	11	61.111
1	11	61.111
0	11	61.111
0	11	61.111
0	11	61.111
1	11	61.111
1	11	61.111
0	11	61.111
0	11	61.111
1	11	61.111
0	11	61.111
1	11	61.111
1	11	61.111
2	11	61.111
1	11	61.111
1	11	61.111
1	11	61.111
2	11	61.111
0	11	61.111
2	11	61.111
1	11	61.111
1	11	61.111
1	11	61.111
2	11	61.111
2	11	61.111
1	11	61.111
2	11	61.111
10	11	61.111
10	11	61.111
9	11	61.111
10	11	61.111
10	11	61.111
9	11	61.111
11	11	61.111
11	11	61.111
11	11	61.111
10	11	61.111
10	11	61.111
10	11	61.111
11	11	61.111
0	10	55.556
0	10	55.556
0	10	55.556

0	10	55.556
0	10	55.556
0	10	55.556
1	10	55.556
1	10	55.556
0	10	55.556
0	10	55.556
1	10	55.556
0	10	55.556
2	10	55.556
0	10	55.556
0	10	55.556
0	10	55.556
0	10	55.556
0	10	55.556
0	10	55.556
0	10	55.556
1	10	55.556
2	10	55.556
0	10	55.556
0	10	55.556
1	10	55.556
2	10	55.556
2	10	55.556
1	10	55.556
9	10	55.556
9	10	55.556
9	10	55.556
9	10	55.556
9	10	55.556
10	10	55.556
10	10	55.556
9	10	55.556
10	10	55.556
9	10	55.556
10	10	55.556
9	10	55.556
10	10	55.556
0	9	50.000
0	9	50.000
0	9	50.000
0	9	50.000
0	9	50.000
0	9	50.000
1	9	50.000
0	9	50.000
0	9	50.000
1	9	50.000
0	9	50.000
0	9	50.000
0	9	50.000
1	9	50.000
1	9	50.000
0	9	50.000
1	9	50.000
2	9	50.000
0	9	50.000
0	9	50.000
1	9	50.000

0	9	50.000
1	9	50.000
0	9	50.000
1	9	50.000
1	9	50.000
1	9	50.000
1	9	50.000
2	9	50.000
8	9	50.000
8	9	50.000
7	9	50.000
8	9	50.000
8	9	50.000
9	9	50.000
7	9	50.000
8	9	50.000
9	9	50.000
8	9	50.000
9	9	50.000
9	9	50.000
9	9	50.000
9	9	50.000
8	9	50.000
8	9	50.000
7	9	50.000
7	9	50.000
8	9	50.000
9	9	50.000
7	9	50.000
9	9	50.000
0	8	44.444
0	8	44.444
0	8	44.444
0	8	44.444
0	8	44.444
0	8	44.444
0	8	44.444
0	8	44.444
0	8	44.444
0	8	44.444
0	8	44.444
0	8	44.444
0	8	44.444
0	8	44.444
0	8	44.444
0	8	44.444
1	8	44.444
0	8	44.444
0	8	44.444
0	8	44.444
0	8	44.444
0	8	44.444
0	8	44.444
1	8	44.444
0	8	44.444
2	8	44.444
0	8	44.444
0	8	44.444
3	8	44.444

7	8	44.444
7	8	44.444
8	8	44.444
7	8	44.444
7	8	44.444
7	8	44.444
8	8	44.444
8	8	44.444
7	8	44.444
7	8	44.444
7	8	44.444
7	8	44.444
7	8	44.444
8	8	44.444
8	8	44.444
8	8	44.444
8	8	44.444
8	8	44.444
8	8	44.444
8	8	44.444
8	8	44.444
0	7	38.889
0	7	38.889
0	7	38.889
0	7	38.889
0	7	38.889
0	7	38.889
0	7	38.889
0	7	38.889
0	7	38.889
0	7	38.889
1	7	38.889
0	7	38.889
0	7	38.889
0	7	38.889
0	7	38.889
0	7	38.889
0	7	38.889
0	7	38.889
0	7	38.889
2	7	38.889
7	7	38.889
6	7	38.889
7	7	38.889
7	7	38.889
5	7	38.889
7	7	38.889
7	7	38.889
7	7	38.889
6	7	38.889
6	7	38.889
7	7	38.889
7	7	38.889
6	7	38.889
7	7	38.889
7	7	38.889
6	7	38.889
6	7	38.889
7	7	38.889

7	7	38.889
7	7	38.889
7	7	38.889
7	7	38.889
7	7	38.889
7	7	38.889
0	6	33.333
0	6	33.333
0	6	33.333
0	6	33.333
0	6	33.333
0	6	33.333
0	6	33.333
0	6	33.333
0	6	33.333
0	6	33.333
0	6	33.333
0	6	33.333
0	6	33.333
1	6	33.333
0	6	33.333
1	6	33.333
1	6	33.333
0	6	33.333
5	6	33.333
5	6	33.333
5	6	33.333
5	6	33.333
6	6	33.333
6	6	33.333
6	6	33.333
6	6	33.333
6	6	33.333
6	6	33.333
6	6	33.333
6	6	33.333
6	6	33.333
5	6	33.333
6	6	33.333
6	6	33.333
5	6	33.333
5	6	33.333
5	6	33.333
6	6	33.333
6	6	33.333
6	6	33.333
6	6	33.333
0	5	27.778
0	5	27.778
0	5	27.778
0	5	27.778
0	5	27.778
0	5	27.778
0	5	27.778
0	5	27.778
0	5	27.778
0	5	27.778
0	5	27.778
0	5	27.778

0	5	27.778
0	5	27.778
0	5	27.778
0	5	27.778
0	5	27.778
0	5	27.778
1	5	27.778
5	5	27.778
4	5	27.778
4	5	27.778
4	5	27.778
5	5	27.778
5	5	27.778
5	5	27.778
5	5	27.778
5	5	27.778
5	5	27.778
5	5	27.778
5	5	27.778
5	5	27.778
5	5	27.778
5	5	27.778
5	5	27.778
0	4	22.222
0	4	22.222
0	4	22.222
0	4	22.222
0	4	22.222
0	4	22.222
0	4	22.222
0	4	22.222
0	4	22.222
1	4	22.222
0	4	22.222
1	4	22.222
0	4	22.222
0	4	22.222
0	4	22.222
0	4	22.222
0	4	22.222
0	4	22.222
0	4	22.222
0	4	22.222
0	4	22.222
0	4	22.222
0	4	22.222
0	4	22.222
0	4	22.222
0	4	22.222
4	4	22.222
4	4	22.222
4	4	22.222
4	4	22.222
3	4	22.222
4	4	22.222
4	4	22.222
4	4	22.222
4	4	22.222
4	4	22.222

4	4	22.222
4	4	22.222
4	4	22.222
4	4	22.222
0	3	16.667
0	3	16.667
0	3	16.667
0	3	16.667
0	3	16.667
0	3	16.667
0	3	16.667
0	3	16.667
0	3	16.667
0	3	16.667
1	3	16.667
0	3	16.667
1	3	16.667
0	3	16.667
0	3	16.667
1	3	16.667
0	3	16.667
1	3	16.667
1	3	16.667
1	3	16.667
1	3	16.667
0	3	16.667
2	3	16.667
0	3	16.667
0	3	16.667
3	3	16.667
3	3	16.667
3	3	16.667
3	3	16.667
3	3	16.667
3	3	16.667
2	3	16.667
3	3	16.667
2	3	16.667
2	3	16.667
3	3	16.667
3	3	16.667
3	3	16.667
3	3	16.667
3	3	16.667
3	3	16.667
3	3	16.667
3	3	16.667
3	3	16.667
2	3	16.667
3	3	16.667
3	3	16.667
3	3	16.667
2	3	16.667
3	3	16.667
3	3	16.667
3	3	16.667
3	3	16.667
3	3	16.667
0	2	11.111

0	2	11.111
0	2	11.111
0	2	11.111
0	2	11.111
0	2	11.111
0	2	11.111
0	2	11.111
0	2	11.111
0	2	11.111
0	2	11.111
0	2	11.111
0	2	11.111
0	2	11.111
0	2	11.111
0	2	11.111
0	2	11.111
0	2	11.111
0	2	11.111
0	2	11.111
0	2	11.111
0	2	11.111
0	2	11.111
0	2	11.111
0	2	11.111
0	2	11.111
0	2	11.111
2	2	11.111
2	2	11.111
2	2	11.111
2	2	11.111
2	2	11.111
2	2	11.111
2	2	11.111
2	2	11.111
2	2	11.111
2	2	11.111
2	2	11.111
2	2	11.111
2	2	11.111
2	2	11.111
1	2	11.111
2	2	11.111
2	2	11.111
2	2	11.111
2	2	11.111
2	2	11.111
2	2	11.111
2	2	11.111
1	2	11.111
2	2	11.111
2	2	11.111
2	2	11.111
2	2	11.111
2	2	11.111
2	2	11.111
2	2	11.111
2	2	11.111
2	2	11.111
2	2	11.111

2	2	11.111
2	2	11.111
2	2	11.111
2	2	11.111
2	2	11.111
2	2	11.111
2	2	11.111
0	1	5.556
0	1	5.556
0	1	5.556
0	1	5.556
0	1	5.556
0	1	5.556
0	1	5.556
0	1	5.556
0	1	5.556
0	1	5.556
0	1	5.556
0	1	5.556
0	1	5.556
0	1	5.556
0	1	5.556
0	1	5.556
0	1	5.556
0	1	5.556
0	1	5.556
1	1	5.556
0	1	5.556
0	1	5.556
0	1	5.556
0	1	5.556
0	1	5.556
0	1	5.556
0	1	5.556
0	1	5.556
0	1	5.556
1	1	5.556
0	1	5.556
0	1	5.556
0	1	5.556
0	1	5.556
0	1	5.556
0	1	5.556
0	1	5.556
0	1	5.556
0	1	5.556
0	1	5.556
0	1	5.556
0	1	5.556
0	1	5.556
0	1	5.556
0	1	5.556
0	1	5.556
0	1	5.556
0	1	5.556
0	1	5.556
0	1	5.556
0	1	5.556
0	1	5.556

0	1	5.556
0	1	5.556
0	1	5.556
0	1	5.556
0	1	5.556
0	1	5.556
0	1	5.556
0	1	5.556
0	1	5.556
1	1	5.556
0	1	5.556
0	1	5.556
0	1	5.556
0	1	5.556
1	1	5.556
1	1	5.556
1	1	5.556
1	1	5.556
1	1	5.556
1	1	5.556
1	1	5.556
1	1	5.556
1	1	5.556
1	1	5.556
1	1	5.556
1	1	5.556
1	1	5.556
1	1	5.556
1	1	5.556
1	1	5.556
1	1	5.556
1	1	5.556
1	1	5.556
1	1	5.556
1	1	5.556
1	1	5.556
1	1	5.556
1	1	5.556
1	1	5.556
1	1	5.556
0	1	5.556
1	1	5.556
1	1	5.556
1	1	5.556
1	1	5.556
1	1	5.556
1	1	5.556
1	1	5.556
0	1	5.556
1	1	5.556
1	1	5.556
1	1	5.556
1	1	5.556
1	1	5.556
1	1	5.556
1	1	5.556
1	1	5.556
1	1	5.556
1	1	5.556

1	1	5.556
1	1	5.556
1	1	5.556
1	1	5.556
1	1	5.556
1	1	5.556
1	1	5.556
1	1	5.556
1	1	5.556
1	1	5.556
1	1	5.556
1	1	5.556
1	1	5.556
1	1	5.556
1	1	5.556
1	1	5.556
1	1	5.556
1	1	5.556
1	1	5.556
1	1	5.556
1	1	5.556
1	1	5.556
1	1	5.556
1	1	5.556
1	1	5.556
1	1	5.556
1	1	5.556
1	1	5.556
1	1	5.556
1	1	5.556
1	1	5.556
1	1	5.556
1	1	5.556
1	1	5.556
1	1	5.556
1	1	5.556
0	0	0.000
0	0	0.000
0	0	0.000
0	0	0.000
0	0	0.000
0	0	0.000
0	0	0.000
0	0	0.000
0	0	0.000
0	0	0.000
0	0	0.000
0	0	0.000
0	0	0.000
0	0	0.000
0	0	0.000
0	0	0.000
0	0	0.000
0	0	0.000
0	0	0.000
0	0	0.000
0	0	0.000
0	0	0.000
0	0	0.000

0	0	0.000
0	0	0.000
0	0	0.000
0	0	0.000
0	0	0.000
0	0	0.000
0	0	0.000
0	0	0.000
0	0	0.000
0	0	0.000
0	0	0.000
0	0	0.000
0	0	0.000
0	0	0.000
0	0	0.000
0	0	0.000
0	0	0.000
0	0	0.000
0	0	0.000
0	0	0.000
0	0	0.000
0	0	0.000
0	0	0.000
0	0	0.000
0	0	0.000
0	0	0.000
0	0	0.000
0	0	0.000
0	0	0.000
0	0	0.000
0	0	0.000
0	0	0.000
0	0	0.000
0	0	0.000
0	0	0.000
0	0	0.000
0	0	0.000
0	0	0.000
0	0	0.000
0	0	0.000
0	0	0.000
0	0	0.000
0	0	0.000
0	0	0.000
0	0	0.000
0	0	0.000
0	0	0.000
0	0	0.000
0	0	0.000
0	0	0.000
0	0	0.000
0	0	0.000
0	0	0.000
0	0	0.000
0	0	0.000
0	0	0.000
0	0	0.000
0	0	0.000
0	0	0.000

0	0	0.000
0	0	0.000
0	0	0.000
0	0	0.000
0	0	0.000
0	0	0.000
0	0	0.000
0	0	0.000
0	0	0.000
0	0	0.000
0	0	0.000
0	0	0.000
0	0	0.000
0	0	0.000
0	0	0.000
0	0	0.000
0	0	0.000
0	0	0.000
0	0	0.000
0	0	0.000
0	0	0.000
0	0	0.000
0	0	0.000
0	0	0.000
0	0	0.000
0	0	0.000
0	0	0.000
0	0	0.000
0	0	0.000
0	0	0.000
0	0	0.000
0	0	0.000
0	0	0.000
0	0	0.000
0	0	0.000
0	0	0.000
0	0	0.000
0	0	0.000
0	0	0.000
0	0	0.000
0	0	0.000
0	0	0.000
0	0	0.000
0	0	0.000
0	0	0.000
0	0	0.000
0	0	0.000
0	0	0.000
0	0	0.000
0	0	0.000
0	0	0.000
0	0	0.000
0	0	0.000
0	0	0.000
0	0	0.000
0	0	0.000
0	0	0.000
0	0	0.000
0	0	0.000

0	0	0.000
0	0	0.000
0	0	0.000
0	0	0.000
0	0	0.000
0	0	0.000
0	0	0.000
0	0	0.000
0	0	0.000
0	0	0.000
0	0	0.000
0	0	0.000
0	0	0.000
0	0	0.000
0	0	0.000
0	0	0.000
0	0	0.000
0	0	0.000
0	0	0.000
0	0	0.000
0	0	0.000
0	0	0.000
0	0	0.000
0	0	0.000
0	0	0.000
0	0	0.000
0	0	0.000
0	0	0.000
0	0	0.000
0	0	0.000
0	0	0.000
0	0	0.000
0	0	0.000
0	0	0.000
0	0	0.000
0	0	0.000
0	0	0.000
0	0	0.000
0	0	0.000
0	0	0.000
0	0	0.000
0	0	0.000
0	0	0.000
0	0	0.000
0	0	0.000
0	0	0.000
0	0	0.000
0	0	0.000
0	0	0.000
0	0	0.000
0	0	0.000
0	0	0.000
0	0	0.000
0	0	0.000
0	0	0.000
0	0	0.000
0	0	0.000
0	0	0.000
0	0	0.000

0	0	0.000
0	0	0.000
0	0	0.000
0	0	0.000
0	0	0.000
0	0	0.000
0	0	0.000
0	0	0.000
0	0	0.000
0	0	0.000
0	0	0.000
0	0	0.000
0	0	0.000
0	0	0.000
0	0	0.000
0	0	0.000
0	0	0.000
0	0	0.000
0	0	0.000
0	0	0.000
0	0	0.000
0	0	0.000
0	0	0.000
0	0	0.000
0	0	0.000
0	0	0.000
0	0	0.000
0	0	0.000
0	0	0.000
0	0	0.000
0	0	0.000
0	0	0.000
0	0	0.000
0	0	0.000
0	0	0.000
0	0	0.000
0	0	0.000
0	0	0.000
0	0	0.000
0	0	0.000
0	0	0.000
0	0	0.000
0	0	0.000
0	0	0.000
0	0	0.000
0	0	0.000
0	0	0.000
0	0	0.000
0	0	0.000
0	0	0.000
0	0	0.000
0	0	0.000
0	0	0.000
0	0	0.000
0	0	0.000

color legend	remark
	>=75%
	>=50% , <75%
	>=25%, <50%
	>=0%, <25%
