# Supplementary material for: Complex Behavior of ALDH1A1 and IGFBP1 in Liver Metastasis from a Colorectal Cancer
Source: PLoS One. 2016 May 6;11(5):e0155160. doi: 10.1371/journal.pone.0155160 (PMC4859559; doi:10.1371/journal.pone.0155160)
Supplement: S3 Table — (PDF) [file pone.0155160.s006.pdf]

Genes	Average fold-count (log_2)	*p* -value	Patient count_2-fold.upreg
HULC	7.110014914	4.71869E-72	16
COLEC11	4.693084634	9.434E-52	16
MBL2	9.631673707	1.0133E-102	15
CFHR2	9.233561013	1.3846E-103	15
CYP4A11	9.087745669	1.3385E-109	15
ACSM2B	8.867224074	7.05921E-98	15
APOF	8.585492489	6.7431E-100	15
IGFBP1	8.526850893	1.0884E-110	15
ACSM2A	8.40099595	1.89435E-88	15
CFHR1	7.901049177	1.71305E-90	15
SLC25A47	7.721720613	4.56813E-89	15
CYP8B1	7.683404706	7.03741E-87	15
FTCD	7.585255957	2.30162E-83	15
CFHR3	7.453849414	8.201E-86	15
APOC1P1	7.411447954	8.63625E-69	15
HGFAC	7.400657726	1.30703E-79	15
C19orf80	6.923822306	3.76057E-73	15
CCL16	6.360925556	5.73516E-63	15
HSD17B13	6.344763476	4.72965E-71	15
SLC38A3	6.033892733	1.49019E-63	15
AMDHD1	4.405429395	3.1682E-43	15
MARCO	3.696925914	8.1528E-34	15
APOE	2.847386648	9.64467E-23	15
ACSL1	1.876769805	9.40365E-13	15
PRODH2	10.45299796	7.43614E-95	14
CFHR5	10.12060992	6.9752E-110	14
FCN2	9.372428863	3.11972E-86	14
APOA5	8.900869772	7.8448E-100	14
UGT1A4	8.839752501	4.91597E-88	14
CD5L	8.446057729	9.33554E-92	14
CPN2	8.135863979	1.0968E-90	14
CFHR4	7.752500118	8.55989E-71	14
C5orf27	6.551056841	1.1035E-65	14
GCKR	6.26874127	1.18837E-69	14
INHBE	5.384028367	1.026E-54	14
ABCG5	4.587969406	1.07639E-40	14
OGDHL	4.104351312	3.27782E-41	14
TTC36	3.857967212	6.98573E-32	14
ASPDH	3.82378906	9.87635E-33	14
ABCB4	3.422083126	5.09014E-30	14
CBS	2.84257187	7.45393E-23	14
F10	2.815195593	7.42456E-23	14
ATF5	1.945399486	6.87562E-14	14
SLC17A2	10.18921453	7.80512E-91	13
CYP4A22	10.00851757	7.27596E-91	13
UGT3A1	9.524586197	3.72264E-87	13
CYP1A2	8.575277437	5.47536E-85	13
ACOT12	8.442196923	1.66636E-78	13
RTP3	7.566177574	5.68153E-58	13
ACMSD	6.440506414	1.68808E-57	13
THRSP	6.290040867	8.44952E-59	13
PRG4	5.548321676	3.47953E-51	13
AGMO	3.640570984	6.69632E-31	13
CDH2	2.935506558	2.26191E-23	13
ALPL	2.661990792	5.28926E-20	13
TTPA	1.993738301	1.74696E-13	13
CLEC1B	7.173721755	5.91824E-58	12
LOC100507203	6.458996198	1.08335E-61	12

LOC255167	5.974686108	1.02961E-58	12
ABCG8	4.872092774	1.28043E-41	12
SHBG	4.871236821	3.99741E-40	12
PPP1R1A	4.156411501	8.94094E-32	12
SLC6A1	3.206191633	5.45686E-26	12
SLC6A12	2.946365793	1.05384E-22	12
FNDC4	2.381536454	3.98395E-16	12
GLTPD2	2.164768905	5.56461E-15	12
PC	1.871472032	3.35338E-12	12
PLGLA	12.44811825	2.87609E-59	11
GDF2	9.83827282	1.50252E-83	11
APOA4	7.977596211	4.35189E-65	11
UGT1A3	5.154384523	1.01416E-34	11
SARDH	3.413107732	3.51482E-30	11
SLC7A2	2.296812184	7.44442E-15	11
CYP1B1	2.179163689	2.4004E-13	11
LEPR	2.152320326	2.20005E-14	11
ALDH1A1	1.738217681	3.007E-10	11
ENPEP	1.676395882	4.21645E-10	11
RND1	1.571333528	2.5221E-09	11
CYB5A	1.233442981	9.72097E-07	11
FAM99A	10.33742636	4.63982E-80	10
SRD5A2	9.93653373	1.65613E-78	10
FGF21	8.560656534	8.70428E-60	10
SLC22A10	7.928908021	4.53246E-67	10
ART4	4.248424919	1.11048E-35	10
SLC47A1	2.803543799	4.78745E-22	10
ACSS3	2.223845893	3.17728E-14	10
CETP	1.952559611	3.85585E-12	10
CYP27A1	1.85319621	1.08477E-11	10
LCAT	1.728658535	4.31688E-10	10
CYP2D7P1	1.59493288	2.16458E-09	10
ERRFI1	1.41189985	2.25357E-08	10
GPD1	1.394982262	1.28142E-07	10
FABP3	1.372755342	4.00556E-07	10
SHMT1	1.300816509	2.86418E-07	10
FGGY	1.285840092	5.35326E-07	10
PXDC1	1.215970564	4.78705E-06	10
SNX10	1.180155059	3.22888E-06	10

Patient count_2-fold.downregı	Patient count_2-fold.ALL	Patient_ratio (%)
0	16	88.889
0	16	88.889
0	15	83.333
0	15	83.333
0	15	83.333
0	15	83.333
0	15	83.333
0	15	83.333
0	15	83.333
0	15	83.333
0	15	83.333
0	15	83.333
0	15	83.333
0	15	83.333
0	15	83.333
0	15	83.333
0	15	83.333
0	15	83.333
0	15	83.333
0	15	83.333
0	15	83.333
0	15	83.333
0	15	83.333
0	15	83.333
0	14	77.778
0	14	77.778
0	14	77.778
0	14	77.778
0	14	77.778
0	14	77.778
0	14	77.778
0	14	77.778
0	14	77.778
0	14	77.778
0	14	77.778
0	14	77.778
0	14	77.778
0	14	77.778
0	14	77.778
0	14	77.778
0	14	77.778
0	14	77.778
0	14	77.778
0	13	72.222
0	13	72.222
0	13	72.222
0	13	72.222
0	13	72.222
0	13	72.222
0	13	72.222
0	13	72.222
0	13	72.222
0	13	72.222
0	13	72.222
0	13	72.222
0	13	72.222
0	12	66.667
0	12	66.667

0	12	66.667
0	12	66.667
0	12	66.667
0	12	66.667
0	12	66.667
0	12	66.667
0	12	66.667
0	12	66.667
0	12	66.667
0	11	61.111
0	11	61.111
0	11	61.111
0	11	61.111
0	11	61.111
0	11	61.111
0	11	61.111
0	11	61.111
0	11	61.111
0	11	61.111
0	11	61.111
0	11	61.111
0	10	55.556
0	10	55.556
0	10	55.556
0	10	55.556
0	10	55.556
0	10	55.556
0	10	55.556
0	10	55.556
0	10	55.556
0	10	55.556
0	10	55.556
0	10	55.556
0	10	55.556
0	10	55.556
0	10	55.556
0	10	55.556
0	10	55.556
0	10	55.556

color legend	remark
(orange)	>=50% , <75%
(blue)	>=25%, <50%
