# Supplementary material for: Complex Behavior of ALDH1A1 and IGFBP1 in Liver Metastasis from a Colorectal Cancer
Source: PLoS One. 2016 May 6;11(5):e0155160. doi: 10.1371/journal.pone.0155160 (PMC4859559; doi:10.1371/journal.pone.0155160)
Supplement: S4 Table — (PDF) [file pone.0155160.s007.pdf]

S4 Table. Primers and conditions for real time RT-PCR in the 9 selected genes

Genes	Primer sequence 5'-3'	Tm, ℃	Size, bp
ALDH1A1	*Forward:* CGGGAAAAGCAATCTGAAGAGGG	60	147
	Reverse: GATGCGGCTATACAACACTGGC		
ERRFI1	*Forward:* TGAGGAAGACCTACTGGAGCAG	60	111
	Reverse: GTATTAGGCGCTCCTGAGCAGA		
IGFBP1	*Forward:* TCCTTTGGGACGCCATCAGTAC	60	134
	Reverse: GATGTCTCCTGTGCCTTGGCTA		
CCL16	*Forward:* GTGTTGCCAAGGAGACTAGTGG	60	125
	Reverse: TCTTGGACCCAGTCGTCATTGG		
ATF5	*Forward:* GCTCGTAGACTATGGGAAACTCC	60	133
	Reverse: CATCCAGTCAGAGAAGCCATCAC		
PRG4	*Forward:* TGTGACTGCGACGCCCAATGTA	60	135
	Reverse: GGTTTGAGATGCTCCTGAAGGTG		
CDH2	*Forward:* CGC GTG AAG GTT TGC CAG T	60	181
	Reverse: GTT TGG CCT GGC GTT CTT TAT		
HGFAC	*Forward:*GAA TCC CTC ACC AGA GTC CA	62	112
	Reverse: GCA GGA ACG TCC TCT TCT TG		
INHBE	*Forward:* CCCAGAATAACTCATCCTCCACC	62	163
	Reverse: GGACAGGTGAAAAGTGAGCAGG		
